# Reactive Radical Etching of Quartz by Microwave Activated CH_4_/H_2_ Plasmas Promotes Gas Phase Nanoparticle Formation

**DOI:** 10.1021/acs.jpca.4c05787

**Published:** 2024-12-10

**Authors:** Michael N. R. Ashfold, Basile F. E. Curchod, Daniel Hollas, Jie Ma, Yuri A. Mankelevich

**Affiliations:** 1 School of Chemistry, University of Bristol, Bristol BS8 1TS, U.K.; 2 School of Physics, Sun Yat-sen University, Guangzhou 510275, China; 3 State Key Laboratory of Optoelectronic Materials and Technologies, Sun Yat-sen University, Guangzhou 510006, China; 4 Skobeltsyn Institute of Nuclear Physics, Moscow State University, Leninskie gory, Moscow 119991, Russia

## Abstract

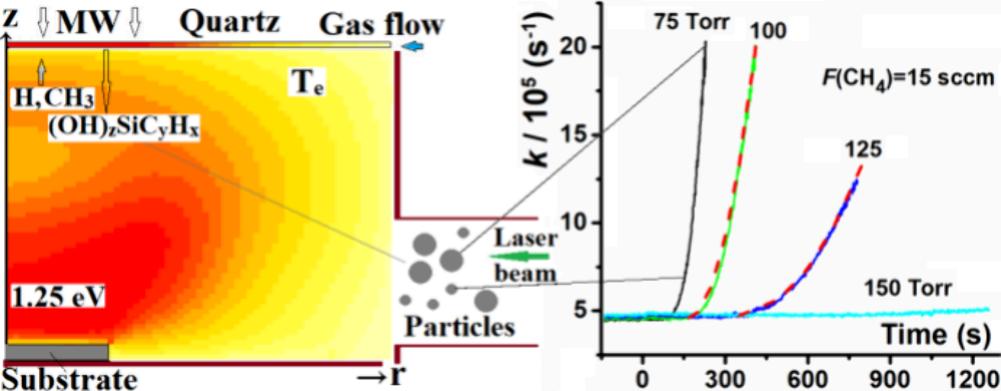

An attenuation of visible probe radiation identified in earlier absorption studies of microwave plasma-activated CH_4_/H_2_/Ar gas mixtures is shown to arise from nanoparticles in under-pumped regions on opposing sides of a reactor used for diamond chemical vapor deposition. The present modeling studies highlight (i) ejection of Si-containing species into the gas phase by reactive radical etching of the quartz window through which the microwave radiation enters the reactor, enabled by suitably high window temperatures (*T*_SiO2_) and the synergistic action of near-window H atoms and C_*y*_H_*x*_ radicals; (ii) subsequent processing of the ejected material, some of which are transported to and accumulate in stagnation regions in the entrance to the reactor side arms; and (iii) the importance of Si in facilitating homogeneous gas phase nucleation, clustering, and nanoparticle growth in these regions. The observed attenuation, its probe wavelength dependence, and its variations with changes in process conditions can all be rationalized by a combination of absorption and scattering contributions from Si/C/H containing nanoparticles with diameters *d* in the range of 50–100 nm. Possible implications for Si incorporation in CVD diamond samples are discussed.

## Highlights

1. 2D(*r*, *z*) self-consistent modeling of microwave plasma-activated CH_4_/H_2_ gas mixtures for a range of pressures and CH_4_ input mole fractions.
2. Proposed reactive radical etching mechanism for SiO_2_ when exposed to microwave activated C/H gas mixtures.
3. Model and experimental demonstrations of the role of etched Si in nucleating growth of larger molecules/clusters/nanoparticles in microwave plasma-activated C/H gas mixtures.
4. Demonstration and rationalization of homogeneous gas phase nanoparticle growth in stagnation volumes within a reactor used for diamond chemical vapor deposition.
5. Electronic absorption data for small SiC_*y*_H_*x*_ species, with potential relevance to interpretations of some diffuse interstellar bands.

## 1 Introduction

The reactive ion etching (RIE) of silicon (Si), quartz (SiO_2_), and related solid substrates has been much studied in recent decades, reflecting the importance of the process in microelectronics and other technologies.^1,2^ Energetic ions (with energies in the range of a few tens to hundreds of electron volts) incident on a solid surface cause bond breakage. The etched material is then released following reaction with radical species present in the low pressure (typically up to tens of mTorr) plasma-activated gas mixture. Unwanted and unintended etching of the walls of industrial plasma reactors operating under soft plasma conditions, with the assistance of much lower energy ions (<10–20 eV), is a related issue of ongoing interest, given its role as a source of contaminant in “clean” plasma processing.^3,4^

Studies of the etching of SiO_2_ substrates at elevated temperatures (*T*_SiO2_ > 1000 K) in a microwave (MW) activated hydrogen plasma operating at much higher (tens of Torr) pressures have been reported recently.^5,6^ The measured increase in etching rate (ER) with increasing *T*_SiO2_ was parametrized in terms of an effective activation energy *E*_a_ = 126 ± 8 kJ mol^–1^, but it is important to note that other factors must also contribute to this reported *E*_a_ value. Increasing the pressure (the means by which *T*_SiO2_ was increased in the reported experiments) also changes the incident H atom flux. Thus, this *E*_a_ value does not describe the pure temperature dependence of the etching rate; the *E*_a_ value for the etching process under a constant incident H atom flux should be much lower. The role of H atoms in etching SiO_2_ is discussed schematically in the work of Zhou et al.^4^ Negative or weak temperature dependences for ER(*T*) have been reported for SiO_2_ when exposed to H_2_ plasmas in the form of a direct current (DC) hollow discharge^7^ and as an inductively coupled plasma.^4^ The latter study proposed a SiO_2_ erosion mechanism, initiated by H atoms inserting into Si–O–Si linkages and forming surface SiH and SiOH groups. This mechanism is based on earlier *ab initio* modeling results,^8^ which demonstrated that H atoms can break strained Si–O bonds in continuous amorphous silicon dioxide (a-SiO_2_) networks and return energy barriers for this process in the range of 0.5–1.3 eV. Analysis of the published results, however, suggests that etching of quartz solely by thermal H atoms (without the assistance of ions and/or plasma radiation) will be minimal at *T*_SiO2_ values close to room temperature. A mechanism involving reactions of H (and O) atoms with Si–CH_*x*_ and Si–H groups has also been developed for plasmas sustained in He/H_2_ (and Ar/O_2_) gas mixtures interacting with porous SiOCH (a popular low-*k* material comprising porous SiO_2_ with methyl groups lining the pores).^9^

Early studies of homoepitaxial diamond film growth by MW plasma-activated chemical vapor deposition (CVD) on a pre-existing diamond sample positioned on a quartz substrate identified Si incorporation, the extent of which could be moderated by adding O_2_ to the input gas mixture.^10^ Many groups have since noted possible etching of a quartz window^11−13^ (or a quartz bell jar or dome^14−16^) in MW plasma-activated chemical vapor deposition (CVD) reactors used for diamond deposition. In the case of the Bristol CVD reactor, we have seen no indication of SiO_2_ etching when using an H_2_ (or an H_2_/Ar) plasma, even at the lowest pressures at which the reactor is deemed safe to operate (*p* ∼ 75 Torr), under which conditions the plasma has expanded closer to the quartz window at the top of the reactor through which the MW radiation enters. Upon adding a few % CH_4_ or C_2_H_2_ to a pre-existing ∼7% Ar in H_2_ plasma, however, particularly at low pressures (*p* ∼75 to 125 Torr), cavity ring down spectroscopy (CRDS) measurements revealed an uncharacterized background attenuation (BA) that developed when probing through the hot plasma region, after an apparent induction time of some tens to hundreds of seconds.^17^ The measurable difference in CRDS experiments performed with a pulsed laser is the ring down time, which decreases as light at the chosen excitation wavelength is lost from the cavity. Whether this deduced loss of light (attenuation) arises from absorption, scattering, or a combination of both will be determined later in this article.

We recently suggested that this BA might be associated with organosilicon products that arise from etching of the quartz window and subsequent processing of these volatile products in the reactor volume.^18^ This suggestion was based on two-dimensional (2D) plasma-chemical modeling results that revealed progressive increases in both the H atom flux near the window surface and the window temperature as the process pressure is reduced. The 2D modeling also revealed the formation of a secondary plasma region at low pressure (*p* = 60 Torr) near the quartz window, with plasma parameters similar to those in the main plasma core centered above the substrate.^18^ However, the etching mechanism prevailing under the specific reactor conditions was not considered in that work nor was the relationship between the volatile etching products and the observed BA, the observed variations in BA with process parameters, or the likely identities of the species responsible for the BA.

Here, we explore these issues in much greater detail. In contrast to the much studied RIE process, we propose a synergistic reactive radical etching (RRE) mechanism for an SiO_2_ surface under process conditions relevant for diamond CVD, in which incident thermal H atoms induce Si–O–Si bond scissions, and reactions with C_*y*_H_*x*_ radicals enable Si–C bond formation and limit Si–O–Si bond reformation. Possible organosilicon compounds are considered as volatile products from the etching process, and precursors for the species responsible for the observed BA are discussed alongside observed variations in the BA with changes in the process parameters. We note that this search for potential species under the prevailing process conditions might impact on the longstanding search for potential carriers of the elusive diffuse interstellar bands (DIBs),^19^ for which organosilicon compounds have been suggested as possible candidates.^20−22^ We also note possible synergies with recent demonstrations of successful growth diamond in liquid metal, at atmospheric pressure, wherein Si is suggested to play an important part in stabilizing tetravalently bonded carbon clusters involved in the earliest nucleation phase.^23^

The remainder of this article is structured as follows. Section 2 summarizes experimental observations relating to the BA as a function of process conditions in the Bristol MW CVD reactor. Section 3 reports 2D modeling results for MW activated C/H gas mixtures, which return plasma parameters and spatial distributions for many species of interest, as functions of (particularly) the input methane mole fraction, *X*_0_(CH_4_), and total pressure, *p*. Armed with these data, the remaining sections present the proposed RRE mechanism for SiO_2_ under the prevailing conditions, consider gas phase processing of the etched material en route to the region probed by CRDS, assess the likely importances of molecular absorption and of scattering and absorption by nanoparticles to the measured BA, before concluding by briefly considering the possible wider implications for diamond growth by CVD from MW activated CH_4_/H_2_ gas mixtures in reactors featuring any quartz or other silicon-containing surfaces.

## 2 Observed Variations in the BA with Changes in Pressure and Methane Mole Fraction

The MW CVD reactor, the laser system, and the optical arrangements for spatially resolved CRDS measurements as functions of height (*z*) above the substrate on which diamond growth occurs have all been described previously.^11^ The CH_4_ (and on occasion C_2_H_2_), H_2_, and Ar source gases were introduced through separate, calibrated mass flow controllers, mixed prior to entering the reactor through two diametrically opposed inlets located close below the quartz window (which constitutes the top of the reactor volume) and exhausted through the base of the reactor. The applied MW power was held fixed at *P* = 1.5 kW throughout, as was the total flow rate, *F*_total_ = 565 sccm (standard cubic centimeters per minute), of which *F*(Ar) = 40 sccm and *F*(H_2_) were adjusted to compensate for changes in *F*(CH_4_). Measurements were taken in the pressure range of 75 ≤ *p* ≤ 150 Torr. The reactor was equipped with two side arms, at the ends of which appropriate high reflectivity mirrors were held in kinematic mounts. The intermirror separation *L* = 85 cm. The side arms, which were mounted symmetrically about slot apertures (29 mm (vert) by 7.8 mm (horiz)) on opposing sides of the reactor (aluminum walls, ∼23 mm thick, with internal water cooling channels), comprised a rigid length (4 cm) of stainless steel tube and then an edge welded bellows section (20 cm compressed length) followed by a further length of a stainless steel tube that terminated with the mirror mount. The bottom of the slot apertures was designed to be ∼2 mm below (and thus to allow unimpeded sight of) the top surface of the substrate. The outer lengths of the side arms were held in rigid clamps, and the incorporated bellows allowed the column sampled by the CRDS measurements to be translated vertically, without loss of cavity alignment and with submillimeter precision, relative to the fixed reactor volume. The internal diameters of all elements in the side arms were no less than 38 mm. The slot apertures between the reactor volume and the side arms mean that the side arms were under-pumped.

The measurable of interest is the change in the ring down rate, Δ*k* (s^–1^), at the chosen probe wavelength, λ. Δ*k* is directly proportional to the loss of probe beam intensity on passing along the column separating the highly reflective mirrors, *i.e*., to the difference in absorbance along (or scattering from) the probed column when the attenuating species is present relative to when it is not.

**Figure 1:**
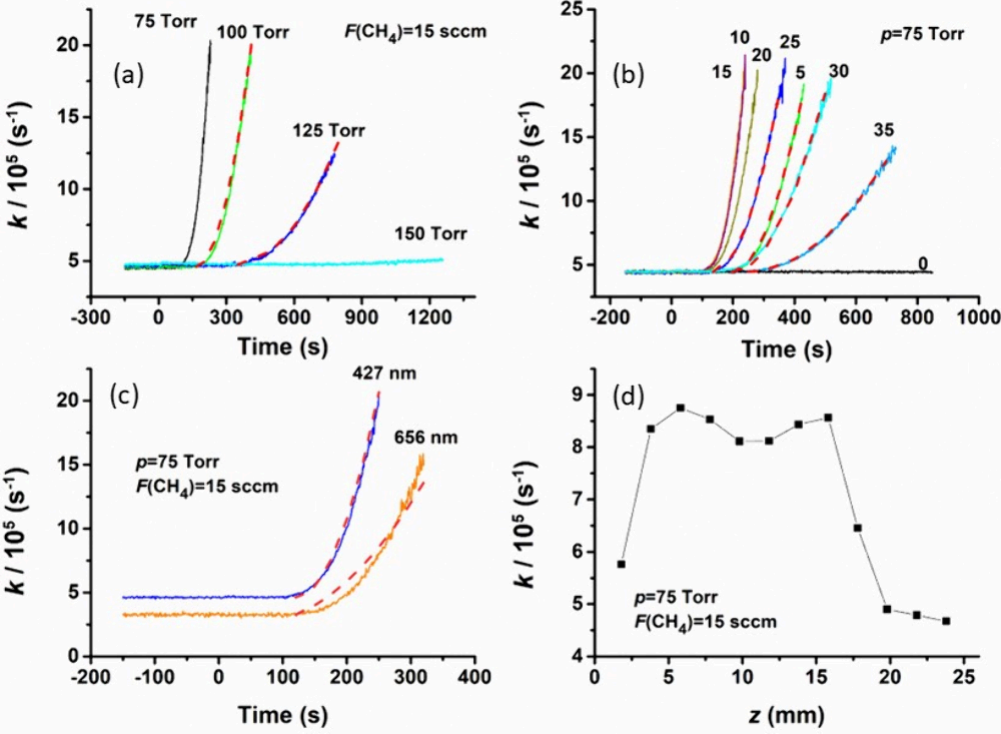
Plots showing (a) the measured ring down rate, *k*, measured at λ = 427 nm as a function of time after introducing *F*(CH_4_) = 15 sccm to a pre-existing H_2_/Ar plasma (at *t* = 0) operating at *P* = 1.5 kW and four different total pressures; (b) variations in the *k* vs *t* dependences measured at λ = 427 nm when different *F*(CH_4_) flow rates are introduced to a pre-existing H_2_/Ar plasma (at *t* = 0) operating at *P* = 1.5 kW and *p* = 75 Torr; (c) *k* vs *t* dependences measured at two different probe wavelengths, λ = 427 and 656 nm, when adding *F*(CH_4_) = 15 sccm to a pre-existing H_2_/Ar plasma (at *t* = 0) operating at *P* = 1.5 kW and *p* = 75 Torr; (d) estimated *z*-profile of the BA feature measured when adding *F*(CH_4_) = 25 sccm to a pre-existing H_2_/Ar plasma operating at *P* = 1.5 kW and *p* = 75 Torr. The various red dashed curves in panels (a–c) show sample fits to the experimental data, using eq 45 together with the *t*_ind_ (= *t – t*_1_) and *b* values reported in Section 6.2.2.

Several of the key observations pertaining to the BA of interest are listed in Figure 1. Figure 1a shows the ring down rate *k* measured at λ = 427 nm, along the column at *z* = 10 mm above the substrate surface, as a function of time after introducing *F*(CH_4_) = 15 sccm to a pre-existing H_2_/Ar plasma operating at *P* = 1.5 kW at three different total pressures. This wavelength is close to, but off-resonance from, the well-documented CH(A–X) absorption band, enabling measurements with the same mirrors and a ring down cavity alignment optimized by such CH column density studies.^11^ The CH_4_ flow was introduced (and *F*(H_2_) was adjusted in a compensatory manner) at *t* = 0. The *k* value at negative *t* is determined by the mirror reflectivity at this wavelength. Companion optical emission spectroscopy (OES) measurements^17,24,25^ showed that the H-Balmer-α emission intensity from the hot plasma region increased (reflecting the increase in electron density upon introducing some CH_4_ to the pre-existing H_2_/Ar plasma) and that the C_2_(d–a) and CH(A–X) emissions appeared and had all attained their new asymptotic values by *t* ∼ 2 s, emphasizing the rapidity of process gas mixing and plasma modification under the prevailing conditions. However, the BA monitored at λ = 427 nm developed much more slowly: both the apparent induction period and the subsequent rate of growth were sensitively dependent on *p*. The growth in ring down rate at later times showed an approximately quadratic time dependence. Measurement stopped when the ring down rate had become too fast to determine with the available nanosecond laser pulse durations. The BA emerged and grew most quickly at the lowest *p*.

As Figure 1b shows, the induction period and the subsequent rate of growth of BA were also very sensitive to *F*(CH_4_). Given *P* = 1.5 kW, *p* = 75 Torr, and *F*_total_ = 565 sccm, the species responsible for the BA appeared in the probed column most quickly and built up fastest at *F*(CH_4_) ∼ 15 sccm. Repeat experiments showed that the induction times and subsequent BA growth rates measured for any given combination of *p* and *F*(CH_4_) values were relatively insensitive to the immediate prior conditions under which the reactor had operated. Figure 1c illustrates similar behavior when probing at λ = 656 nm, off-resonant from the H-Balmer-α transition. The smaller *k* value at *t* < 0 in this case reflects the higher reflectivity of the mirrors available at the longer probe wavelength. As Figure 1c shows, the induction times under any given process conditions appear similar at both wavelengths, but the subsequent rate of increase in *k* is slower when probing at λ = 656 nm. Other key observations are that the BA could be flushed away by introducing a pulse of argon into the extremities of the side arms, but it persisted (almost indefinitely) if the plasma was extinguished and the gas flow and the pumping shut off. Note that no BA was discerned when operating this reactor under the then-prevailing “base” conditions for diamond CVD, *i.e*., *P* = 1.5 kW, *p* = 150 Torr, *F*(CH_4_) ∼ 25 sccm, *F*(Ar) = 40 sccm, and *F*(H_2_) = 500 sccm.^11^

Spatially- (*z-*) resolved H(*n* = 2) atom and CH and C_2_ radical column densities were obtained by translating the CRDS probe beam relative to the substrate surface.^11^ Determining the spatial profile of the BA was not straightforward, however, as its associated *k* never reached an asymptotic value. The best-estimated spatial distribution shown in Figure 1d was obtained as follows: The run started by measuring *k* at a position far above the substrate (*z* = 24 mm) and then translating the probe column in 2 mm steps to progressively smaller *z* and measuring *k* over a fixed time interval (Δ*t*, just a few s) at each value until it was nearing the substrate surface. The process was continued but now stepped to larger *z*. Clearly, *k* at any *z* is increasing with time but, as the other panels in Figure 1 show, the late time variation of *k*(*z*) varies sufficiently linearly with *t* that we simply averaged the *k* values determined at any given *z* on the incoming and outgoing stages of the run. Necessarily, considerable uncertainty attaches to the data shown in Figure 1d, but the uncertainty is not sufficient to hide the key finding that the spatial profile of the BA appears relatively flat in the central region and decreases steeply at small and large *z*. Qualitatively, at least, its spatial profile might be viewed as mimicking that of the hot plasma region. However, we also note that the extremities of the profile come quite close to the top and bottom of the slot apertures in the reactor wall and that we can exclude any physical blocking of the CRDS beam since this would reduce the circulating light intensity and thus manifest as an obvious increase in Δ*k*.

## 3 2D Modeling Results for MW Activated C/H Gas Mixtures under Relevant Process Conditions

Two series of calculations for different *p* and *X*_0_(CH_4_) in a CH_4_/H_2_ mixture were performed using the 2D(*r*, *z*) self-consistent model that has been tested, enhanced and verified against absorption and OES data for many different MW activated gas mixtures including H_2_,^26^ Ar/H_2_ and Kr/H_2_,^27^ CH_4_/H_2_,^24,28,29^ N_2_/H_2_ and N_2_/CH_4_/H_2_,^30,31^ and SiH_4_/H_2_ and SiH_4_/CH_4_/H_2_^32^ plasmas operating in the Bristol MW CVD reactor. Note that the gas mixture used in the original experiments contained some Ar (*X*_0_(Ar) ∼ 7%) to enable actinometry studies.^17,25^ Modeling and experimental studies of the effects of Ar additions to CH_4_/H_2_ gas mixtures operating under the same reactor conditions have shown that such low Ar fractions have minimal effect on the plasma parameters,^27,33^ and the present modeling thus does not include Ar or Ar-related processes. The radius and height (the distance between the top surface of the reactor base plate and the lower surface of the quartz window that defines the top of the cylindrical reactor) are *R* = 61 and *H* = 61 mm, respectively, and the substrate is a 3 mm-thick, 32 mm diameter disk seated centrally on a thin spacer wire on the base plate. The specific blocks of the model address the plasma-chemical and electron (e) kinetics, heat, mass, and species transfer and gas-surface interactions and solve Maxwell’s equations to calculate the spatially dependent MW electromagnetic (EM) fields and MW power. The electron energy distribution functions for all cells in the (*r*, *z*) grid are calculated from the Boltzmann equation and sets of e-H, e-H_2_, e-C_*y*_H_*x*_, and e-ion collision cross sections (for different local mixture compositions, reduced electric fields, and gas temperatures, *T*_g_) to provide the necessary rate coefficients for the blocks describing the plasma-chemical kinetics and the EM fields. *r*, *z* = 0 defines the center of the top surface of the substrate. The full reaction mechanism involves 34 species and ∼300 reactions, including key neutral species (H(*n* = 1–3), H_2_, C, CH, ^3^CH_2_, ^1^CH_2_, CH_3_, CH_4_, C_2_H_*x*_ (*x* = 0–6), C_*y*_H_*x*_ (*y* = 3 and 4, *x* = 0–2), selected excited electronic states of H_2_, C(^1^D) atoms, CH(A^2^Δ), C_2_(d^3^Π_g_), and C_2_(a^3^Π_u_) radicals, electrons, and the following ions: H^+^, H_2_^+^, H_3_^+^, C_2_H_2_^+^, C_2_H_3_^+^, and C_4_H_3_^+^. Key reaction mechanisms, plasma parameters, and deposition processes were reprised recently^18,34^ and in two earlier papers.^30,31^

The H atom loss probability, γ_H_, on a quartz surface is very dependent on the process conditions. Values ranging from 10^–5^ (ref (35)) up to 0.02 (ref (36)) have been reported. Here, we have adopted a constant value γ_H_ = 0.003 from a study by Gubarev et al.^37^ involving similar H atom fluxes to those in the present work on SiO_2_ walls. Test calculations showed that reducing this chosen γ_H_ value has minimal effect on the predicted H atom concentrations, while increasing it by an order of magnitude causes a <3-fold reduction in the predicted H atom concentrations near the quartz window.

The calculation of the thermal balance for the 7 mm-thick quartz window has also been refined, introducing heat extraction coefficients for the cooling of the upper surface (by the fan-driven forced air flow)^38^ and due to thermal emission from both window surfaces (assuming a quartz emissivity ∼0.75). To set the scene, we first note that ∼20% of the total absorbed MW power is expended in window heating under the peak BA conditions. Details of the partitioning of *P* into other channels (∼14% to the substrate, ∼38% to the base plate, and ∼28% to the sidewalls of the cylindrical reactor) are presented in ref (18). The contribution from energy released in gas-surface reactions (mainly H atom adsorption followed by hydrogen recombination) is negligible (∼0.5% *cf.*, thermal conduction term). The two window heat loss terms, which are of comparable magnitude for the central window area, are balanced by ∼80% of the incident heat gained by conduction from the hot plasma region. The remaining ∼20% from this source is dissipated in the reactor walls, via radial conduction through the quartz window. The temperature-dependent conduction coefficient for quartz is modeled as λ_SiO2_ = 0.0169 + 9 × 10^–6^ × (*T*_SiO2_ – 600) W cm^–1^ K^–1^, from ref (39). This approach results in temperature drops of ∼180 ± 10 K across the window thickness at *r* = 0 (*i.e., T*_SiO2_ ∼890 ± 10 K and ∼710 ± 5 K for the lower and upper (cooled) surfaces, respectively, under the conditions of present interest with *p* = 75 Torr). Convective heat transfer between the internal window surface and the flowing C/H gas is not included in the present modeling. At the low (typically <1 cm s^–1^) radial gas velocities just beneath the window, convective heat transfer is estimated to be 2 orders of magnitude lower than the calculated average heat transfer via thermal conduction (∼2.5 W cm^–2^ for the peak BA regime). We also note that calculations with normal (*i.e., g* = 9.81 m s^–2^) and zero acceleration due to gravity suggest that buoyancy effects have little impact on the prevailing plasma parameters, consistent with conclusions reached in the earlier study of Prasanna et al.^40^

**Figure 2:**
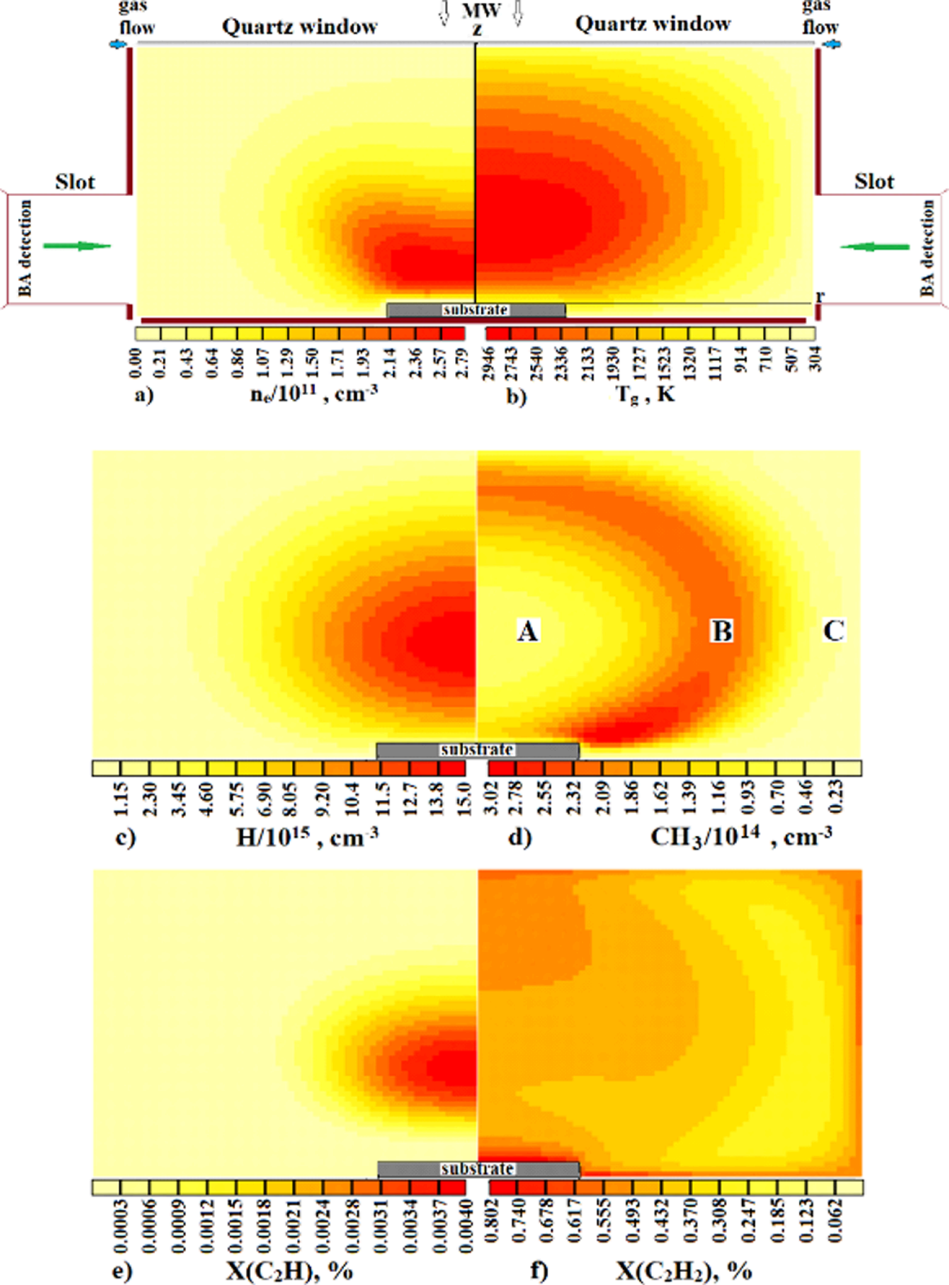
Plots showing the calculated 2D(*r*, *z*) distributions (left or right half only) of selected key quantities in a MW plasma-activated 2.66% CH_4_ in the H_2_ gas mixture (equivalent to *F*(CH_4_) = 15 sccm in *F*_total_ = 565 sccm) operating at *p* = 75 Torr and *P* = 1.425 kW. (a) Electron concentration, *n*_e_ (in cm^–3^), (b) gas temperature, *T*_g_ (in K), (c, d) H atom and CH_3_ radical concentrations, [H] and [CH_3_], respectively, in units of cm^–3^, (e, f) mole fractions *X*(C_2_H) and *X*(C_2_H_2_), in %. The substrate diameter, *d*_s_ = 32 mm, temperature, *T*_s_ = 900 K, and the color scale used in each panel span 13 equal intervals. Key elements of the reactor (substrate, quartz window, slots, the incident MW radiation, the gas inlets, and the vertical range over which the BA is probed by CRDS) are illustrated in panels (a, b), which also define the *r* and *z* axes used in the modeling with the origin (*r* = 0, *z* = 0) at the top center of the substrate. Necessarily therefore, the sections through the reactor volume appear smaller in panels (a, b), *cf*. the other four panels, but the false color plots in all panels display the same 0 ≤ *r* ≤ 61 mm and 0 ≤ *z* ≤ 61 mm ranges. The three main zones of hydrocarbon conversion are indicated as **A**, **B,** and **C** in panel (d).

Figure 2 shows spatial distributions for (a) the electron density, *n*_e_, and (b) the gas temperature, *T*_g_, returned by 2D(*r*, *z*) modeling of MW activated 2.66% CH_4_ in H_2_ plasma (*i.e.*, equivalent to *F*(CH_4_) ∼ 15 sccm in *F*_total_ = 565 sccm) operating at *p* = 75 Torr and an absorbed power *P* = 1.425 kW. As previously stated, the modeling shows a hot plasma region (**A** in Figure 2d) centered over the substrate. The plasma volume is larger than that in equivalent C/H plasmas operating at higher pressures, but the degree of ionization is again small (≤10^–6^). *T*_g_(*r* = 0, *z*) decreases from ∼2950 K in the hot plasma core to ∼900 K just below the window center. H atoms, mostly formed by thermal dissociation of H_2_ in region **A**, diffuse throughout the reactor (Figure 2c) and drive H-shifting reactions that ensure that most of the carbon in the CH_4_ source gas is converted to C_2_H_2_. C_2_H_2_ accounts for ∼95% of total carbon in the hot plasma region. The maximal H atom mole fractions, *X*_max_(H), in the 2.66%CH_4_/H_2_ mixture featured in Figure 2 increase from ∼6 to ∼16% across the pressure range *p* = 75–150 Torr. *X*_max_(H) ∼ 6% in the plasma core (*i.e*., at *r* = 0, *z* ∼ 20 mm) at *p* = 75 Torr corresponds to a maximal concentration [H]_max_ ∼ 1.5 × 10^16^ cm^–3^. This value changes little at this *p* as *X*_0_(CH_4_) is varied across the 0.88–6% range.

Figure 2d–f shows the spatial distributions for three carbon-containing species (CH_3_ and C_2_H radicals, and the majority C-containing species C_2_H_2_). For clarity, the first of these data are displayed in terms of species number density, while the latter two distributions are plotted as mole fractions. These panels serve to re-emphasize well-known trends:^24^ H abstraction reactions dominate in region **A** (as exemplified by the localized spatial distribution of C_2_H mole fraction, which is controlled by the C_2_H_2_ + H ↔ C_2_H + H_2_ equilibrium that shifts in favor of products at high *T*_g_), whereas H addition reactions (aided by a third body) typically dominate at lower *T*_g_, as illustrated by the spatial distributions of the CH_3_ number density (which maximizes in the annular region **B**) and the C_2_H_2_ mole fraction (which maximizes in the peripheral regions **C** near the cooled reactor walls).

**Figure 3:**
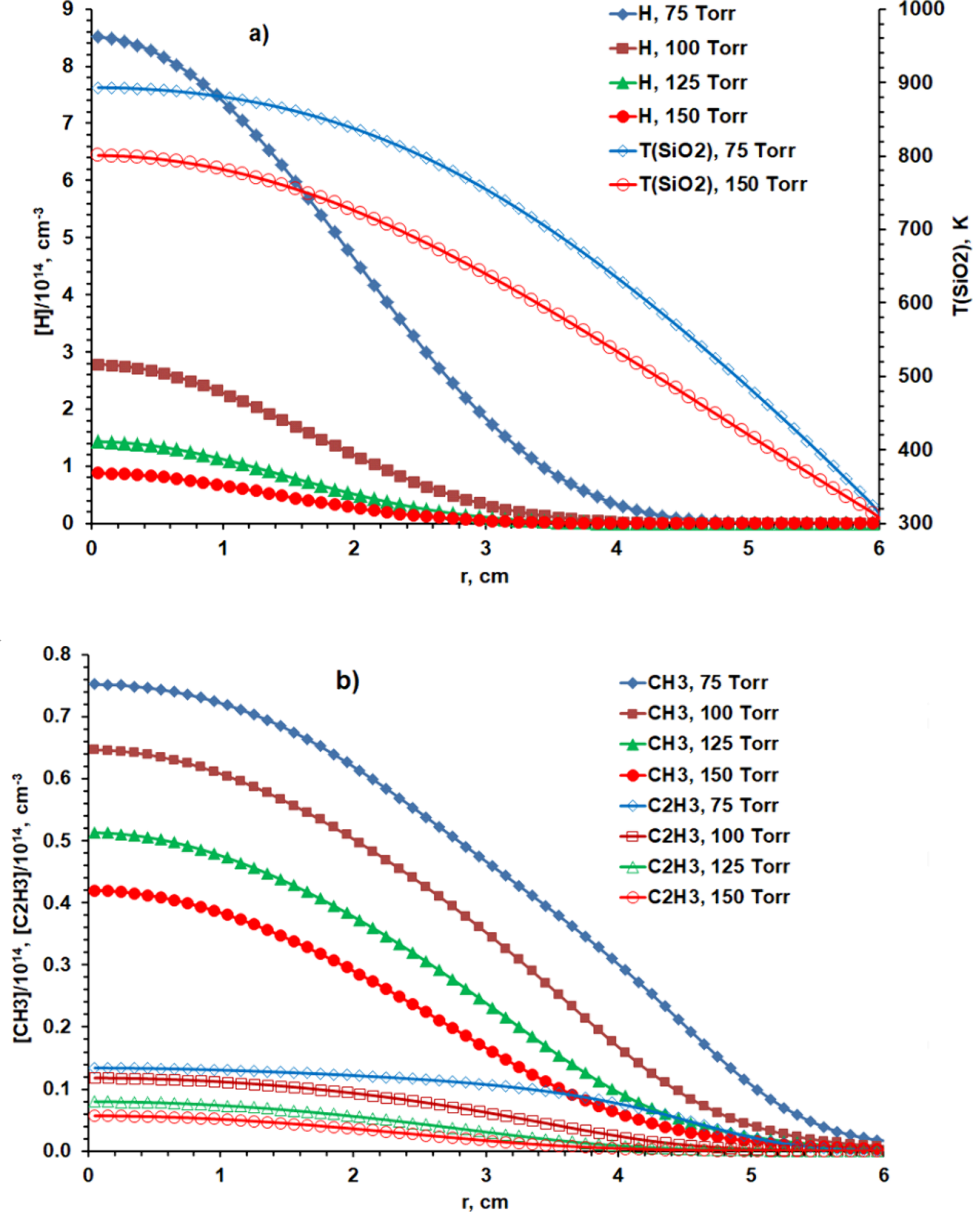
Radial concentration distributions of (a) H atoms and (b) CH_3_ and C_2_H_3_ radicals close below the quartz window for four pressures in the range 75 ≤ *p* ≤ 150 Torr at *X*_0_(CH_4_) = 2.66% (*F*(CH_4_) ∼ 15 sccm) and *P* = 1.425 kW. The temperature of the underside of the quartz window *T*_SiO2_(*r*) is also shown for *p* = 75 and 150 Torr in panel (a).

**Figure 4:**
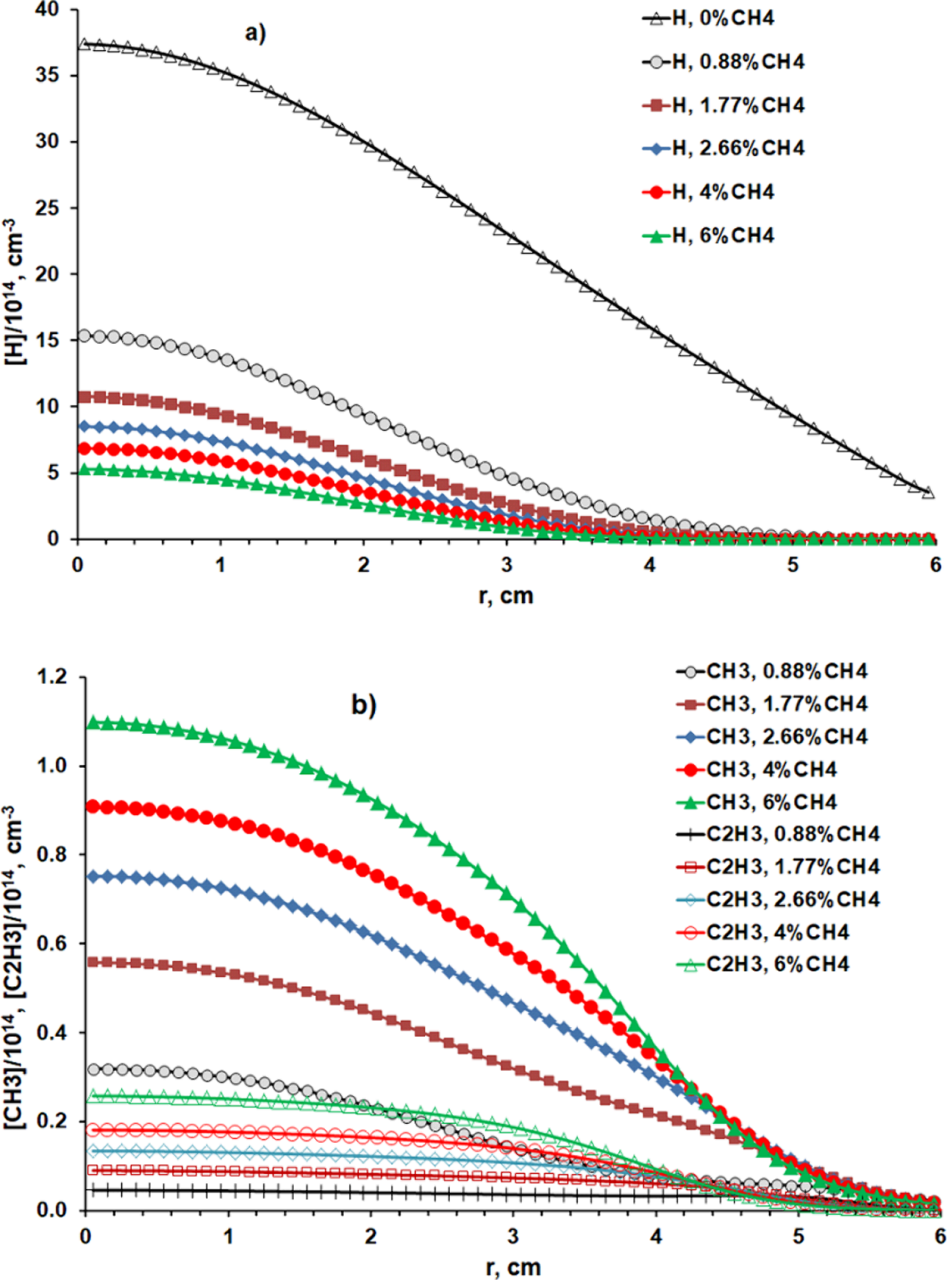
Radial concentration distributions of (a) H atoms and (b) CH_3_ and C_2_H_3_ radicals close below the quartz window for five CH_4_ input mole fractions in the range 0.88 ≤ *X*_0_(CH_4_) ≤ 6% (corresponding to (*F*(CH_4_) ∼5, 10, 15, 22.5, and 34 sccm) at *p* = 75 Torr and *P* = 1.425 kW. Panel (a) also includes the corresponding [H](*r*) profile for *X*_0_(CH_4_) = 0.

**Figure 5:**
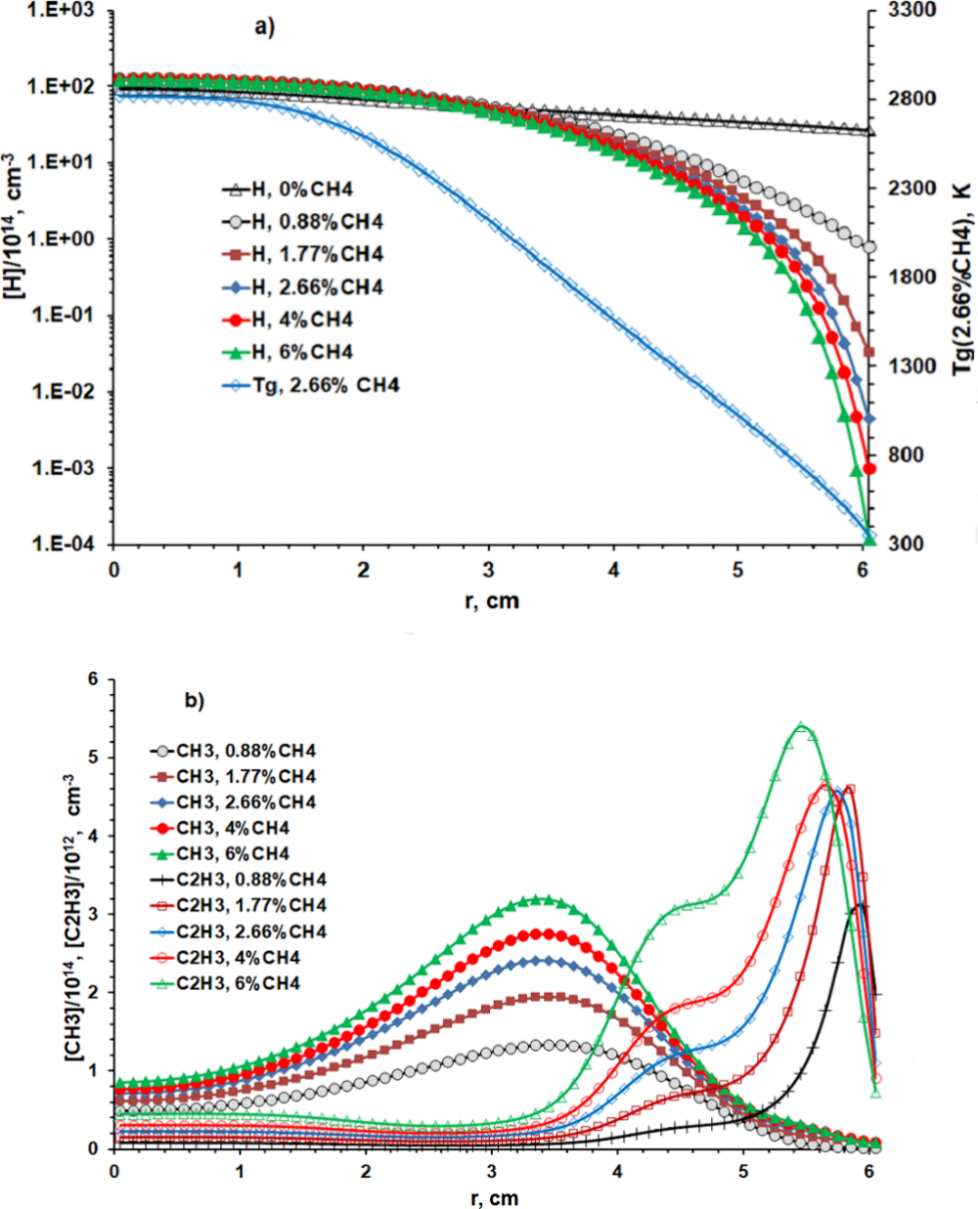
Radial concentration distributions of (a) H atoms and (b) CH_3_ and C_2_H_3_ radicals along the column probed at *z* = 10 mm for five CH_4_ input mole fractions in the range 0.88 ≤ *X*_0_(CH_4_) ≤ 6% (corresponding to (*F*(CH_4_) ∼5, 10, 15, 22.5, and 34 sccm) at *p* = 75 Torr and *P* = 1.425 kW. Panel (a) also includes the corresponding [H](*r*) profile for *X*_0_(CH_4_) = 0 and the *T*_g_(*r*) profile in the case when *X*_0_(CH_4_) = 2.66%.

The parameters varied in the experiment were *p* and *F*(CH_4_), and the present focus is the possible etching of the SiO_2_ window and how this might be revealed by optical measurements along a column centered at *z* ∼ 10 mm. The remainder of this section reports outputs from the 2D(*r*, *z*) modeling that inform on both issues. Figures 3 and 4 report *r*-profiles for selected key parameters and species concentrations at *z* = 58 mm, just below the quartz window, while Figure 5 reports *r*-distributions for many of the same quantities at *z* = 10 mm, near the middle of the range of *z* values probed in the CRDS experiments.

Figure 3 shows the *r*-dependent number density distributions of (a) H atoms and (b) CH_3_ and C_2_H_3_ radicals, henceforth, [H], [CH_3_], and [C_2_H_3_], at *z* = 58 mm (*i.e.*, just below the quartz window) at *p* = 75, 100, 125, and 150 Torr. Among these species, the [H] distribution is more tightly peaked around *r* = 0, and [H](*r* = 0) decreases by an order of magnitude on doubling *p*. [CH_3_](*r* = 0) is least affected by increasing *p*, only decreasing from ∼8 × 10^13^ to ∼4 × 10^13^ cm^–3^ as *p* is doubled from 75 to 150 Torr, while [C_2_H_3_](*r* = 0) decreases by a factor of ∼2.5 over this same *p* range. The [CH_3_](*r* = 0)/[C_2_H_3_](*r* = 0) ratio increases weakly (by ∼30%) with increasing *p*. Several competing factors contribute to these differences. The marked decrease in [H] in regions away from the plasma core (including the near reactor wall regions) upon doubling *p* affects the balances within the C_1_H_*x*_ and C_2_H_*x*_ families and the interconversion between them, and the relative contribution from unprocessed source gas in regions away from the plasma core increases as the plasma volume decreases. Figure 3a also shows predicted radial temperature distributions of the underside of the quartz window at *p* = 75 and 150 Torr, which, at the lower *p*, decreases from ∼900 K at *r* = 0 to a little more than 300 K at its outer edge (*r* = 60 mm).

Figure 4 shows the corresponding (a) [H](*r*) and (b) [CH_3_](*r*) and [C_2_H_3_](*r*) distributions just below the quartz window (*z* = 58 mm) calculated for five CH_4_ input mole fractions in the range 0.88 ≤ *X*_0_(CH_4_) ≤ 6% (equivalent to (*F*(CH_4_) ∼5, 10, 15, 22.5, and 34 sccm in *F*_total_ = 565 sccm) at *p* = 75 Torr and *P* = 1.425 kW. Figure 4a also includes the corresponding [H](*r*) profile for *X*_0_(CH_4_) = 0, the values of which are much higher than [H] for all non-zero *X*_0_(CH_4_). Introducing any CH_4_ leads to an obvious narrowing of the [H](*r*) distribution and a decrease in [H](*r* = 0), which is calculated to decrease from ∼3.3 × 10^15^ to ∼4.3 × 10^14^ cm^–3^ upon increasing *X*_0_(CH_4_) from 0 to 6%. The [CH_3_](*r* = 0)/[C_2_H_3_](*r* = 0) ratio just below the quartz window is predicted to decrease from ∼7 to ∼4.3 upon increasing the *X*_0_(CH_4_) from 0.88 to 6%.

Figure 5 shows the corresponding (a) [H](*r*) (and *T*_g_(*r*)) and (b) [CH_3_](*r*) and [C_2_H_3_](*r*) distributions along the column probed at *z* = 10 mm calculated for the same five CH_4_ input mole fractions in the range 0.88 ≤ *X*_0_(CH_4_) ≤ 6% with *F*_total_ = 565 sccm, *p* = 75 Torr, and *P* = 1.425 kW. Introducing CH_4_ reduces the plasma volume. [H](*r* = 0) increases by ∼30%, but [H](*r* = 60 mm) falls dramatically, by more than 5 orders of magnitude when *X*_0_(CH_4_) = 6%. [CH_3_] shows its characteristic radial distribution in such reactors,^24^ maximizing in an annular shell around the hot plasma region (*i.e*., region **B** at *r* ∼ 35 mm for the defined *F*_total_, *p*, and *P* conditions). The C_2_H_3_ radical concentrations start to grow rapidly at larger *r* and, as Table 1 shows, are comparable to [CH_3_] at *r* = 60 mm when *X*_0_(CH_4_) = 0.88%. Table 1 also reiterates the well-documented trend toward more heavily hydrogenated C_*y*_H_*x*_ species in the periphery of the reactor (region **C** in Figure 2d). Note that this trend is not replicated by organosilicon species because of different thermochemistry. The relatively lower Si–H bond energy (*cf.* that of the C–H bond) ensures different Si-containing species in the cooler regions of MW reactors operating with dilute SiH_4_/CH_4_/H_2_ gas mixtures (*i.e*., Si, *c*-SiC_2_, Si=CH_2_, *etc*., rather than CH_4_ and C_2_H_*x*_, *x* = 2, 4, and 6 species).^32^ The number densities of C_2_H_*x*_ species at *r* = 60 mm, *z* = 10 mm grow more rapidly with increasing *X*_0_(CH_4_) than does [CH_4_]; the CH_3_ and C_2_H_3_ radical densities peak at *X*_0_(CH_4_) =2.66% and 0.88%, respectively. The modeling predicts negligible C_2_H radical density in this region (reflecting the temperature dependence of the C_2_H_2_ + H ↔ C_2_H + H_2_ equilibrium, which strongly favors the reactant side at low *T*_g_).

**Table 1:** H Atom and Selected C_*y*_H_*x*_ Radical and Molecule Concentrations [cm^–3^] at *r* = 60 mm, *z* = 10 mm Returned by the Present Simulations for CH_4_/H_2_ Gas Mixtures at *P* = 1.425 kW, with Different *X*_0_(CH_4_) Operating at *p* = 75 Torr, and with Different *p* Operating at *X*_0_(CH_4_) = 2.66%^a^

X_0_(CH_4_), %	0.88	1.77	2.66	4.00	6.00	2.66	2.66	2.66
*p*, Torr	75	75	75	75	75	100	125	150
[H]	7.71 × 10^13^	3.31 × 10^12^	4.34 × 10^11^	9.83 × 10^10^	1.21 × 10^10^	2.90 × 10^10^	4.59 × 10^9^	1.15 × 10^9^
[CH_3_]	1.17 × 10^12^	7.89 × 10^12^	9.00 × 10^12^	8.45 × 10^12^	7.53 × 10^12^	5.68 × 10^12^	3.64 × 10^12^	2.42 × 10^12^
[CH_4_]	1.86 × 10^16^	3.10 × 10^16^	4.28 × 10^16^	5.17 × 10^16^	6.51 × 10^16^	5.16 × 10^16^	6.27 × 10^16^	8.09 × 10^16^
[C_2_H_2_]	1.05 × 10^15^	4.62 × 10^15^	1.09 × 10^16^	1.92 × 10^16^	4.23 × 10^16^	1.61 × 10^16^	1.96 × 10^16^	2.53 × 10^16^
[C_2_H_3_]	1.97 × 10^12^	1.48 × 10^12^	1.10 × 10^12^	9.11 × 10^11^	7.29 × 10^11^	4.69 × 10^11^	2.14 × 10^11^	1.07 × 10^11^
[C_2_H_4_]	1.67 × 10^14^	1.37 × 10^15^	3.35 × 10^15^	5.70 × 10^15^	1.14 × 10^16^	5.63 × 10^15^	7.18 × 10^15^	9.50 × 10^15^
[C_2_H_5_]	1.41 × 10^11^	1.26 × 10^12^	3.69 × 10^12^	7.06 × 10^12^	1.49 × 10^13^	7.70 × 10^12^	1.02 × 10^13^	1.26 × 10^13^
[C_2_H_6_]	1.45 × 10^14^	5.64 × 10^14^	1.03 × 10^15^	1.34 × 10^15^	1.80 × 10^15^	1.12 × 10^15^	1.27 × 10^15^	1.68 × 10^15^

a The calculated C_2_H concentration was <1 cm^–3^ under all conditions considered here.

## 4 Reactive Radical Etching (RRE) Mechanism for SiO_2_

2D(*r*, *z*) modeling (Section 4) provides the context upon which any proposed mechanism must build, and the experiment (Section 2) provides observations that any such model should seek to accommodate. The experimental measurements reveal a distinct spatial profile for the BA (Figure 1d), and the BA only appears in the probed region many tens of seconds after introducing CH_4_ to a pre-existing H_2_/Ar plasma. Both the induction time between adding CH_4_ and the emergence of the BA, and the subsequent rate of BA increase, are sensitive to *p* and to *F*(CH_4_). The BA emerges earliest and grows fastest at the lowest pressure investigated (*p* = 75 Torr) and at an intermediate CH_4_ flow rate (*F*(CH_4_) = 15 sccm). For simplicity, the phrase “peak BA” is henceforth used to define such conditions. It is important to emphasize at this point that no hydrocarbon-only mechanism appears capable of accommodating the observed *p* and *F*(CH_4_) dependences of the BA, *e.g*., its reduced intensity and increased appearance time at high *F*(CH_4_) and *p*.

The 2D(*r*, *z*) modeling shows that the lowest *p* corresponds to the highest quartz window temperature and the highest near-window H atom concentration. Adding CH_4_ results in substantial near-window C_*y*_H_*x*_ radical concentrations and reduces the local H atom concentration. Under peak BA conditions, the 2D(*r*, *z*) modeling predicts *T*_SiO2_ ∼900 and ∼715 K at the center of the lower and upper surfaces of the window (these values are still far below the melting temperature of quartz, ∼1700 °C), and respective near-window [H], [CH_3_], and [C_2_H_3_] densities of ∼8.5 × 10^14^, ∼7.5 × 10^13^, and ∼1.4 × 10^13^ cm^–3^ at *r* = 0, respectively.

The discussion in this section assumes that the species responsible for the observed BA derive from volatile products formed from etching the SiO_2_ window—either directly or by subsequent gas phase processing of the primary ejecta. Recall that, in the experiments, *F*(CH_4_) was added to a pre-existing Ar/H_2_ plasma, *i.e.*, the quartz window was already hot (*T*_SiO2_ ∼ 890 K at *r* = 0 and *p* = 75 Torr) prior to the addition of any CH_4_. However, the observed BA was not seen to be sensitive to the immediate prior conditions under which the reactor had been operated. Thus, the observed BA behavior is unlikely to be attributable to the microscopic state of the quartz window (lightly etched, more heavily etched, *etc*.) immediately prior to CH_4_ addition.

Figures 3 and 4 show the near-window (*z* = 58 mm) radial concentration profiles for H atoms and CH_3_ and C_2_H_3_ radicals for various *p* and *X*_0_(CH_4_) conditions. The ion concentrations are much lower (<10^9^ cm^–3^), and the energies of these ions (gained via the near wall potential and lost in elastic collisions with gas molecules and atoms) are also low—less than ∼0.3 eV^6^ at the prevailing pressures (*p* ≥ 75 Torr) and electron temperatures (*T*_e_ < 0.5 eV near the window). Incident ions are thus unlikely to make any significant contribution to SiO_2_ etching. The most abundant closed shell molecules (CH_4_ and C_2_H_2_) will be chemically inert at the SiO_2_ surface. Hereafter, therefore, we focus on the possible roles of the radicals with the highest concentrations in the near-window region, *i.e*., H atoms and CH_3_ and C_2_H_3_ radicals. At first glance, the near-window concentrations of these species and their variations with *p* and *X*_0_(CH_4_) have no obvious correlations with the measured BA behavior, but indirect scenarios wherein the primary etched products undergo subsequent plasma processing are discussed in Section 5.

Notwithstanding the fact that any quartz surface in a working microwave CVD reactor will have been subjected to some preconditioning, the simplest SiO_2_ etching mechanism must start with Si–O–Si bond scissions, which, under the prevailing conditions, will most likely be initiated by H atoms. Such bond scission reactions have been simulated for strained Si–O bonds in amorphous SiO_2_^8^ and invoked to explain the observed etching of a quartz substrate in an Ar/H_2_ inductively coupled plasma.^4^ Zhou et al.^4^ used the following reactions from ref (8):
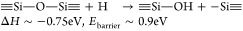
1
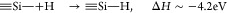
2where the ≡ symbol indicates that the Si atom is bound to three other atoms in the lattice. The passivation of the ≡Si— radical site by process (eq 2), which is relatively much more important than passivation via the endothermic dissociative addition of H_2_, will be in local equilibrium with ≡Si— site restoration via the exothermic H abstraction process (eq 3)
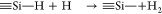
3

Reactions 2 and 3 together constitute the surface-enabled recombination (2H → H_2_) of incident H atoms and a null cycle for the surface. Reaction −1, for which the calculated energy barrier is ∼1.7 eV, and reaction 4 offer possible routes to restoring (“self-healing”) ≡Si—O—Si≡ bridges at higher *T*_SiO2_:^8^
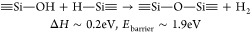
4

Sneh and George^41^ have reported that hydroxylated silica surfaces evolve H_2_O upon heating (420–830 K) via reaction 5
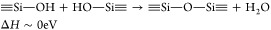
5though we also note that adjacent ≡Si—OH groups are unlikely to be formed in the initial stages of H atom etching of a pristine quartz surface: reactions 1 and 2 only yield ≡Si—H and ≡Si— groups adjacent to a ≡Si—OH group.

The progressive scission of ≡Si—O—Si≡ bonds via reaction 1 (up to four Si–O bonds per Si surface atom, up to six Si–O bonds per Si–O–Si bridge, up to eight Si–O bonds per double Si–O–Si–O–Si bridge, *etc*.) could result in many different volatile products. For single Si groups, we recognize two main product families: H_*x*_Si(OH)_3–*x*_ (*x* = 0–3) radicals and H_*x*_Si(OH)_4–*x*_ (*x* = 0–3) molecules, which are distinguished by the detail of the final surface bond cleavage. Products in the latter family require that the OH group formed in the final bond scission (reaction 1) associates with the departing species. We note that this proposed etching mechanism precludes the formation of SiH_4_ as a primary ejected molecule. We can also anticipate some formation of other radical products, *e.g.,* H_*x*_Si(OH)_2–*x*_ (*x* = 0–2) biradicals. The relative numbers of Si–O (*n*_Si–O_) and Si–H (*n*_Si–H_) bonds in the volatile products leaving the surface (*n*_vp_) are quite predictable: *n*_Si–O_ = 2*n*_vp_ and *n*_Si–H_ = 2*n*_vp_ – *n*_radical_ (where *n*_radical_ is the number of volatile radical species).

Adding CH_4_ enriches the potential chemical complexity but causes a minimal change in *T*_SiO2_. The calculated near-window hydrocarbon radical concentrations (predominantly CH_3_ and C_2_H_3_, Figures 2–4) are expected to disrupt the dynamics of the ≡Si— ↔ ≡Si—H site alteration processes (reactions 2 and 3) by offering rival radical addition processes (eq 6):
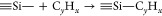
6

Such reactions involving nonradical hydrocarbon species are expected to have much lower rate coefficients, *e.g., k*_6_[cm^3^ s^–1^] = 1.2 × 10^–12^ × exp(−1564/*T*_SiO2_) for C_2_H_4_ addition to trimethylsilyl (CH_3_)_3_Si radicals,^42^ and thus to be less important under the present conditions. The H atom abstraction rate constant for reaction 3 is fast.^43^ Relative to the radical–radical reactions (eq 6), the abstraction reactions of chemisorbed C_*y*_H_*x*_ radicals by H atoms (*i.e.*, ≡Si—CH_3_ + H → ≡Si— + CH_4_ or ≡Si—C_2_H_3_ + H → ≡Si— + C_2_H_4_) are likely to be much slower—both on steric grounds and by analogy with the corresponding gas phase reactions. The greater relative stabilities of the products from the hydrocarbon passivation processes (eq 6) are expected to reduce the possible role of the surface “healing” reaction (eq 4) once CH_4_ has been added to the process gas mixture.

The CH_2_ group in the surface-bound ≡Si—C_2_H_3_ group is unlikely to be etched by reaction with an H atom on account of the high reaction endothermicity (>2.4 eV^44^). In contrast, incident H atoms are expected to etch ≡Si—CH_3_ surface groups by two successive exothermic reactions:
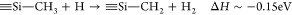
7and
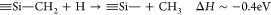
8(where the reaction enthalpies are estimated from thermochemical data reported in ref (44)). This expectation is based, in part, on earlier studies of H_2_ plasma etching of ≡Si—CH_3_ groups in porous SiOCH low-*k* dielectrics at relatively modest film temperatures (*T*_SiOCH_ = 453 K).^45^ Thus, we propose the operation of a synergistic reactive radical etching (RRE) mechanism for SiO_2_ surfaces exposed to MW activated C/H/(Ar) plasmas operating under the conditions of present interest (*T*_SiO2_ ∼ 900 K, and comparable near-window H and C_*y*_H_*x*_ radical concentrations). Incident H atoms break and help restore surface and near-surface ≡Si—O—S≡ bridges, but the presence of C_*y*_H_*x*_ radicals disrupts the restoration reaction, leading to etching and the appearance of various volatile organosilicon products.

Reactions 1–3 and 6–8 can result in families of volatile radicals and closed shell species with respective formulas (C_2_H_3_)_*x*1_(CH_3_)_*x*2_Si(OH)_3–*x*1–*x*2_ and (C_2_H_3_)_*x*1_(CH_3_)_*x*2_Si(OH)_4–*x*1–*x*2_ (*x*1 + *x*2 = 0–3). Such species have been investigated previously.^46−48^ As for the H_2_ plasma processing discussed above, volatile radicals are formed when the last Si–O_surface_ bond breaks to leave the nearby ≡Si—OH group at the surface, while ejection of a volatile molecule leaves the radical ≡Si— site at the surface. Release of many other possible products into the gas phase can be envisaged, *e.g*., (C_*y*_H_*x*_)_*x*1_SiH_*x*2_(OH)_*z*_ (*x*1 + *x*2 + *z* = 3 or 4) species with H atoms instead of C_*y*_H_*x*_ (CH_3_ and/or C_2_H_3_) groups, and more complex species like (C_*y*_H_*x*_)_*x*1_(HO)_*z*1_Si–O–Si(OH)_*z*2_(C_*y*_H_*x*_)_*x*2_. The latter is particularly noteworthy. For simplicity, the discussion to this point outlined reaction sequences focused on one Si atom in a ≡Si—O—Si≡ bridge. However, one can also envisage a more extensive etching scenario, where damage develops at multiple proximal sites, ultimately reaching a point where accumulating stresses (and maybe local surface reconstructions) enable the ejection of much larger moieties.

## 5 Gas Phase Processing of Ejected Organosilicon Species

All products desorbing from the SiO_2_ surface will be further processed within the reactor volume, via myriad gas phase reactions involving H atoms, H_2_ and C_*y*_H_*x*_ molecules, radicals, and even ions. Previous modeling of MW activated dilute SiH_4_/CH_4_/H_2_ gas mixtures^32^ using available thermochemical data^44,49^ identified the preferential gas phase chemical conversion processes for the three simplest Si-containing families in such mixtures, namely, SiH_*x*_ (*x* = 0–4), SiCH_*x*_ (*x* = 0–3), and SiC_2_H_*x*_ (*x* = 0–2), which favored the most stable variants in each family, namely, Si, SiCH_2_, and SiC_2_. The present study involves no SiH_4_ source gas but includes an abundance of more complex, heavy etching products (Si_*m*_O_*z*_C_*y*_H_*x*_, where we acknowledge the possibility of nanoclusters involving multiple Si atoms). Any detailed modeling of the chemical conversions in such a Si/O/C/H mixture would require a massive expansion of the available database. This is not realistic without reliable information about reaction mechanisms, rate coefficients, and thermochemical data for the many possible heavy species. Here, therefore, we simply outline likely basic trends and conversions of the etched organosilicon species prior to seeking explanations for the measured BA behavior under different reactor conditions.

### 5.1 Processing in the Hot Plasma Core

We start by considering the likely conversions of the primary etched species as they migrate along the *r* = 0, *z* axis (mainly by diffusional and thermodiffusional transfer) from the near quartz window region to the detection zone running through the hot plasma core. The (CH_3_)_*x*1_(C_2_H_3_)_*x*2_SiH_*x*3_(OH)_*z*_ (*x*1 + *x*2 + *x*3 + *z* = 3 and 4 and *x*1 + *x*2 + *x*3 < 4) species will be thermally decomposed and modified by reactions analogous to those of the lighter CH_*x*_, C_2_H_*x*_, and SiH_*x*_ families that have been studied previously.^11,24,32^ For reference, we reiterate the axial variation of some of the more important gas parameters under different process conditions (all for *P* = 1.425 kW). At *p* = 150 Torr and *X*_0_(CH_4_) = 2.66%, *T*_g_(*r* = 0, *z*) increases from ∼800 K near the window to ∼3175 K in the plasma core, while the H atom mole fractions increase from ∼0.004% just below the center of the underside of the window to *X*_max_(H) ∼16% in the plasma core. At *p* = 75 Torr and *X*_0_(CH_4_) = 0, *X*(H) increases from ∼0.5% at *r* = 0, *z* = 58 mm to *X*_max_(H) ∼4.5% in the plasma core (where *T*_g_ ∼ 3000 K). The corresponding values for *p* = 75 Torr and *X*_0_(CH_4_) = 6% are *X*(H) ∼ 0.06% and *X*_max_(H) ∼ 6%.^18,24^ Two classes of conversion reactions—H-shifting and H atom abstraction reactions and organosilicon–hydrocarbon coupling reactions—are now considered in turn.

#### 5.1.1 Conversions Driven by H-Shifting and H Atom Abstraction Reactions

The Si–OH bond dissociation energy, *D*(Si–OH) ∼ 5.8 eV, exceeds those of all other bond dissociation energies of current interest, *e.g*., *D*(H_3_Si–H) ∼ 4 eV, *D*((CH_3_)_3_Si–H) ∼ 4.1 eV, *D*(H_3_Si–CH_3_) ∼ 3.9 eV, *D*((CH_3_)_3_Si–CH_3_) ∼ 4.1 eV, *D*((CH_3_)_3_Si–C_2_H_5_) ∼ 4 eV, and *D*((CH_3_)_3_SiO–H) ∼ 5.1 eV.^50^ Nonetheless, OH groups will be progressively lost in the hot and partly dissociated hydrogen gas environment via reaction 9
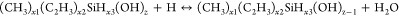
9

The reverse reaction will be unimportant given that [H] ≫ [H_2_O] under the prevailing conditions. In contrast, the loss of H atoms from SiH_*x*3_ groups (reaction 10) will be in balance with the reverse reaction:
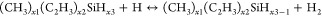
10

Our previous modeling^32^ showed that fast H-shifting reactions ensure that SiH_*x*3_ groups partition in favor of bare Si atoms throughout the whole reactor volume. Henceforth, therefore, we focus the analysis and trace further changes simply in species of the form (CH_3_)_*x*1_(C_2_H_3_)_*x*2_Si (while still recognizing that some Si–OH and Si–H groups could survive in the final products). Further dehydrogenation of these simpler species will occur via H abstraction in H-shifting reactions similar to those prevailing in the CH_*x*_ and C_2_H_*x*_ families,^11,24^*e.g.*,
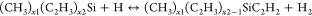
11
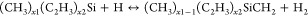
12

Such dehydrogenation processes will open routes to different isomers of these product families. The resulting organosilicon families (from the simplest SiCH_*x*_ species to the most complex SiC_6_H_*x*_ moieties) that might appear in the probed column via reactions 9–12 can be systematized as follows:

##### SiCH_*x*_

Primary sources of this family are etched products of the form (CH_3_)SiH_*x*3_(OH)_*z*_ (*x*3 + *z* = 2 and 3, with *x*3 < 3). Si=CH_2_ will presumably be the most populated SiCH_*x*_ species.^32^ The (lower concentration of) SiCH radicals, which mostly arise via H abstraction from Si=CH_2_—, will maximize in the hot plasma region **A**, with a spatial distribution reminiscent of that of the C_2_H radical (recall Figure 2e) whose concentration profile is similarly determined by the balance of the H-shifting reaction C_2_H_2_ + H ↔ C_2_H + H_2_.

##### SiC_2_H_*x*_

Etched products of the forms (CH_3_)_2_SiH_*x*3_(OH)_*z*_ and (C_2_H_3_)SiH_*x*3_(OH)_*z*_ are likely primary sources for this family. After gas phase processing, the greatest population is likely to be in the form of the thermodynamically favored cyclic SiC_2_ species.^32^

##### SiC_3_H_*x*_

(CH_3_)_3_Si(OH)_*z*_ (*z* = 0 and 1) and (CH_3_)(C_2_H_3_)SiH_*x*3_(OH)_*z*_ (*x*3 + *z* = 1 and 2, with *x*3 < 2) etched products are potential sources for this family. SiC_3_H and SiC_3_ species that feature in the later discussion can both arise via H-shifting reactions involving SiC_3_H_*x*_ (*x* ≥ 2). Note that the Si atom in these and any heavier primary etched species considered here are bonded to multiple C atoms. As such, H-shifting reactions alone could enable formation of cyclic- (rhomboidal) forms of SiC_3_, but isomerization would be required to form the higher lying linear SiC_3_ species.^51^ In similar vein, ground state (linear) SiC_3_H radical formation would require rearrangement of the heavy atoms in the primary etched species, but there should be no such impediment to forming the higher lying cyclic isomer of SiC_3_H, involving a rhomboidal SiC_3_ moiety similar to one of the known forms of SiC_3_ and one C–H bond.^22^

##### SiC_*n*_H_*x*_ (*n* = 4–6)

The parent sources for these families are, respectively, the etched products (C_2_H_3_)_2_SiH_*x*3_(OH)_*z*_ (*x*3 + *z* = 1 and 2, with *x*3 < 2), (CH_3_)(C_2_H_3_)_2_Si(OH)_*z*_ (*z* = 0 and 1), and (C_2_H_3_)_3_Si(OH)_*z*_ (*z* = 0 and 1). The respective SiC_*n*_H_2_ + H ↔ SiC_*n*_H + H_2_ and SiC_*n*_H + H ↔ SiC_*n*_ + H_2_ equilibria will shift toward the product side in the hotter regions, so the axial column density profiles of the various SiC_*n*_H and SiC_*n*_ species would be expected to peak in the plasma region.

##### SiH_*x*_

This family would derive from primary etched products of the form SiH_*x*3_(OH)_*z*_ (*x*3 + *z* = 3 and 4, and 0 ≤ *x*3 ≤ 3), which are likely to be rare under the conditions of interest. The SiH_*x*3_(OH)_*z*_ + H ↔ SiH_*x*3_(OH)_*z*−1_ + H_2_O reactions will lead to production of SiH_*x*_ and SiOH (and SiO) species. As noted previously, H-shifting reactions drive the partitioning among SiH_*x*_ species in favor of Si atoms throughout the whole reactor volume.^32^

Beyond these families of volatile organosilicon SiC_*n*_H_*x*_ species deriving from reactions involving H atoms, we also note a wealth of further possible derivatives, *e.g.*, CH_3_(OH)_*z*_Si=CH_2_, C_2_H_3_(OH)_*z*_Si=CH_2_, *z* = 0 and 1, *etc*., that could arise via reactions with the incident radicals.

#### 5.1.2 Organosilicon–Hydrocarbon Coupling Reactions in the Hot Plasma Region

Many other bimolecular gas phase encounters could further redistribute population between these various families,^20,32^ though such effects may be less important in the present case (where SiO_2_ etching constitutes the silicon source) than when silicon is intentionally added into the input gas mixture (*e.g.*, as SiH_4_ or Si_2_H_6_).

Radical species within the SiH_*x*_ family (including relatively low-lying excited-state Si(^1^D) atoms) can provide additional sources of SiC_*y*_H_*x*_ species via reactions with hydrocarbons.^20,22,32,52^ The simplest coupling reactions involve CH_*x*_ species and Si atoms
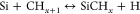
13

SiC_2_H_*x*_ species can also be produced via gas phase reactions involving Si atoms, *e.g.*,
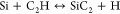
14
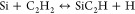
15and H-shifting reactions would ensure a dominance of cyclic SiC_2_ species within the SiC_2_H_*x*_ family.^32^ C_2_H_2_ is a dominant hydrocarbon in most of the reactor volume,^18,24^ so reactions 14 and 15 could provide a route to SiC_2_H_*x*_ (particularly SiC_2_) species. The absolute importance of these reactions under peak BA conditions must be questionable, however, given the expectation that the RRE mechanism results in much higher fluxes of SiC_*y*_H_*x*_ (*cf.* SiH_*x*_) species.

Many bimolecular routes to form SiC_*y*_H_*x*_ (*y* > 2) species can be envisioned. SiC_3_H_*x*_ species, for example, can be expected to arise via both SiCH_*x*1_ + C_2_H_*x*2_ and SiC_2_H_*x*1_ + CH_*x*2_ reactions. Similarly, SiC_4_H_*x*_ products could arise via reactions of SiC_2_H_*x*1_ with C_2_H_*x*2_ species and between SiC_3_H_*x*1_ with CH_*x*2_ species. A short set of potentially more important reactions that should prevail under peak BA conditions includes:
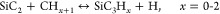
16
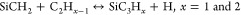
17

As noted previously, SiC_2_ and SiCH_2_ have been identified as being among the more stable species in MW activated SiH_4_/CH_4_/H_2_ gas mixtures, so reactions like reactions 16 and 17 can be expected to contribute throughout much of the reactor volume, with subsequent H-shifting reactions favoring SiC_3_ products. Suggested SiC_4_H_*x*_ production via reactions 18–20, in contrast, can be expected to occur preferentially in region **A**, where the C_2_ and C_2_H radical concentrations are maximal:
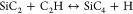
18
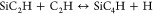
19
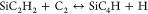
20

Some of these reactions were included in earlier astrophysical models.^20^ The thermochemical and kinetic feasibility of reactions with the (much more abundant) C_2_H_2_ molecule, *e.g*., SiC_2_ + C_2_H_2_ ↔ SiC_4_ + H_2_ and SiC_2_ + C_2_H_2_ ↔ SiC_4_H + H are not known. These reactions are currently not included in the astrophysical models. Production routes via reactions of SiC_*y*_H_*x*_ (*y* > 2) species with hydrocarbons are also not well known and merit additional study.

We note that SiC_3_ has two low-lying rhomboidal forms (labeled *d*-SiC_3_ and *r*-SiC_3_ according to whether the structure includes a transannular C–C or C–Si bond^53^) and a slightly less stable linear form and that SiC_3_ and SiC_4_ (which has a linear ground state geometry) have both been identified in interstellar environments.^54,55^ Some works suggest that both may be present with comparable number densities,^56^ while others predict SiC_3_ to be relatively much more abundant.^52^ SiC_*y*_ (*y* = 3 and 5–8) species have also all been detected in laboratory Fourier transform microwave spectroscopy studies,^57^ and jet-cooled optical spectra have been reported for SiC_2_,^58^ SiCH,^59^ and various SiC_*y*_H (*y* = 3–5)^22^ species.

### 5.2 Processing SiC_*y*_H_*x*_ Species in the Cooler Peripheral Regions

As noted above, previous studies of MW activated dilute SiH_4_/CH_4_/H_2_ gas mixtures have shown that H-shifting reactions favor H-deficient species (*e.g.*, Si atoms, Si=CH_2_ and SiC_2_ radicals, *etc*.) in the hot plasma core.^32^ The Si source in the present environment—SiC_*y*_H_*x*_ species diffusing away from the top quartz window—is very different. The C_*y*_H_*x*_ groups within the etched species will be altered by fast H-shifting reactions (throughout much of the reactor) and three-body H addition reactions (in the cooler regions), depending on the local gas temperature and [H]/[H_2_] ratios, as shown previously for the core C_*y*_H_*x*_ species.^60^ The C_*y*_H_*x*_ groups can also be altered by additional reactions involving Si–C bond breaking and formation, *e.g*.,
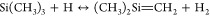
21
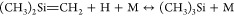
22
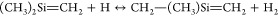
23
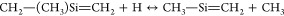
24

These and further similar reactions will lead to a range of organosilicon radical and nonradical species, with different relative abundances and balances in different regions of the reactor. The diversity of organosilicon species will be further expanded by reaction with volatile species containing CH=CH_2_ groups (including the C_2_H_3_ radical itself) and their derivatives. Further, the appearance of organosilicon species with cyclic *c*-SiC_2_ groups or rhombic SiC_3_ groups, *e.g*.,
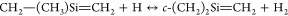
25may also be possible. Further dehydrogenation of the (CH_2_)_2_ group could lead to formation of cyclic *c*-(CH)_2_Si=CH_2_ molecules, containing a C=C double bond, two Si–C bonds, and an Si=C bond. All such species will be dynamically transformed within the reactor, reaching the slot regions with a partitioning appropriate for cool, H-depleted conditions.

Table 1 illustrates such partitioning for the C_*y*_H_*x*_ hydrocarbon species at *r* ∼ 60 mm, *z* ∼ 10 mm (*i.e.*, near the centers of the slots in the reactor wall along the laser probe axis). The calculated CH_3_ radical concentrations (and the [CH_3_]/[CH_4_] ratios) show local maxima at *X*_0_(CH_4_) = 1.77–2.66%, *i.e*., at the *F*(CH_4_) = 10–15 sccm values for which the largest BA is observed. The H atom concentrations in this region decrease rapidly with increasing *X*_0_(CH_4_), from [H] ∼ 8 × 10^13^ cm^–3^ at *X*_0_(CH_4_) = 0.88% down to [H] ∼ 4 × 10^11^ cm^–3^ at *X*_0_(CH_4_) = 2.66% and [H] ∼ 10^10^ cm^–3^ at *X*_0_(CH_4_) = 6%. This hints at the presence of similar maxima in the concentrations of H-rich species like Si(CH_3_)_3_ radicals, CH_2_=Si(CH_3_)_2_ species and, as shown below, chains and clusters derived from such species that include Si–CH_2_–Si bridges and CH_2_/CH_3_/C_2_H_*x*_ end groups.

Reactive volatile species like CH_2_=Si(CH_3_)_2_, less hydrogenated analogues, chains/arrays incorporating methylene bridges (*e.g.*, containing −(CH_3_)_2_Si–CH_2_–Si(CH_3_)_2_– units), and other larger organosilicon species will accumulate in the cool periphery of the reactor, including in the slot regions and, potentially, further along the reactor side arms (bellows) probed by the CRDS experiments. The conditions prevailing in this region favor association of these organosilicon species; rival reactions wherein the radical end groups are depleted by H atom additions are limited by the low H atom concentrations, and loss reactions with H_2_ molecules are limited by the low *T*_g_ values (not much above room temperature). Incoming radical and nonradical organosilicon species are envisaged assembling into chains and/or clusters, via reactions such as
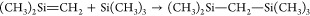
26and
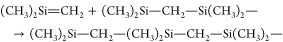
27that have been proposed previously to account for heavier gas phase species detected in the remote plasma CVD of amorphous hydrogenated silicon carbide (a-Si:C:H) films from organosilane sources, and the presence of Si–CH_2_–Si bridges in the as-deposited films.^61,62^ Similar reactions between 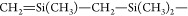
and 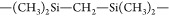
chains constitute chain propagation (heteroclustering) processes, which will be nonterminating given a steady supply of reactive species and chains with CH_2_ end groups.

C_2_H_3_ radicals and, particularly, C_2_H_2_ molecules are also abundant in the cooler peripheral regions (Table 1). C_2_H_3_ radical addition to the Si atom in species such as (CH_3_)_2_Si=CH_2_ could offer a route to chain branching. C_2_H_2_ addition to (CH_3_)_2_Si=CH_2_, forming 1,1-dimethylsilacyclobutene, *i.e*.,
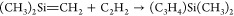
28has also been demonstrated in the gas phase, at *T*_g_ ∼ 920 K,^63,64^ and could offer another route to growing the cluster size (and its C/Si compositional ratio) in the cooler regions. By analogy with reaction 21, it is tempting to suggest a sequence like
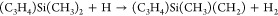
29
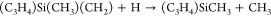
30
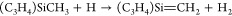
31

The product containing the Si=CH_2_ group is primed to initiate chain growth as per reaction 27, and to react with further C_2_H_*x*_ species, analogues of the additional reaction (reaction 28), to yield
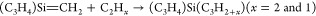
32

The symmetric 4-silaspiro[3.3]hepta-1,5-diene species, containing two C=C double bonds (for *x* = 2) and two four-membered rhomboids with a common Si-node, could be a key “brick” for further clustering/polymerization, without self-limitation, by propagative reproduction of terminal C=C groups and C radical sites. H abstraction from the −CH_2_– or —CH=CH— groups in either ring, *i.e*.,
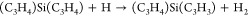
33followed by reactions of 4-silaspiro[3.3]hepta-1,5-diene with these (C_3_H_4_)Si(C_3_H_3_) radicals
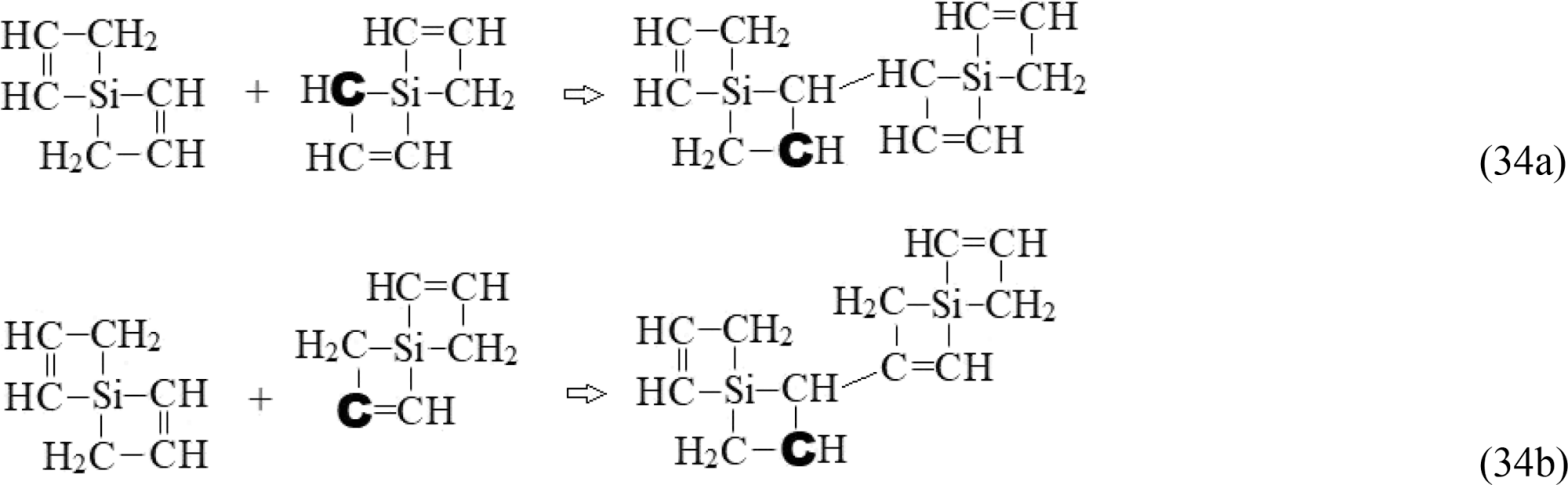
34could constitute initiation steps based on −**C**H– or —**C**= radical sites (marked in bold). Such association reactions, wherein two C–C bonds are formed at the expense of a C=C double bond, have obvious parallels with documented simpler exothermic reactions like H_2_C=CH_2_ + H—**C**=CH_2_ → H_2_C—CH_2_—CH=CH_2_.^65^ A key feature of such reactions is the preservation of a radical site that allows further chain propagation, as illustrated for the −**C**H– site in reaction 35:
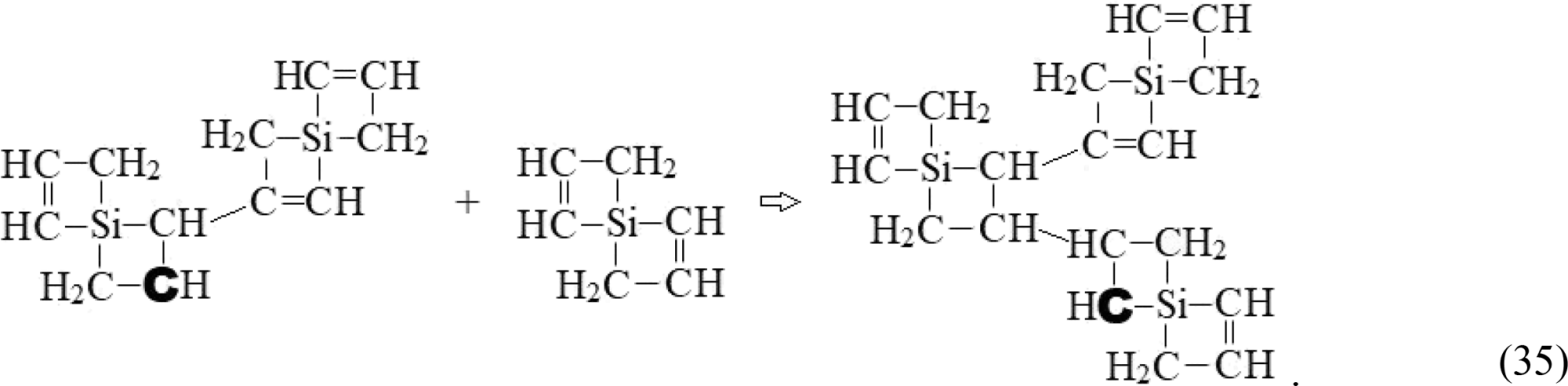
35

Again, we note parallels with the documented exothermic H_2_C=CH_2_ + H–**C**H–CH_3_ → *l*-C_4_H_9_ association reaction.^65^ The products of association reactions of the type illustrated in reaction 35 maintain the requirements (*i.e*., terminal —CH=CH— groups and −**C**H– radical sites) for further clustering via reactions with the “bricks” and larger clusters derived therefrom.

Such initiation and association reactions are not limited to reactions 33–35. At the low *T*_g_ values prevailing in the slot regions, H atom addition to a —CH=CH— group, *e.g.,* (C_3_H_4_)Si(C_3_H_4_) + H → (C_3_H_4_)Si(C_3_H_5_), might well be the most efficient initiator based on a −**C**H– radical site. Note that this does not offer a pure “cold” clustering mechanism at near room temperature, however, since higher *T*_g_ values (>900 K) are required for efficient production of the “bricks” via the temperature-dependent reactions (reactions 28, 29, and 32, for *x* = 2). Further, another H atom addition, *i.e*., (C_3_H_4_)Si(C_3_H_5_) + H → (C_3_H_4_)Si(C_3_H_6_), would serve to repassivate the −**C**H– radical site created by the first addition.

Most of the etched products first appear just below the hot central region of the quartz window, where the reactor conditions are characterized by *T*_g_ > 900 K and high H, CH_3_, and C_2_H_3_ concentrations. As the etched species spread away from the near-window region, they are progressively converted into “bricks” and larger clusters such as those shown in reactions 32, 34, and 35. Nanoparticles (NPs) start to form as the concentrations and sizes of the larger species increase. The efficiencies with which these embryonic NPs grow will vary throughout the reactor volume (and in stagnation regions).

The association of small NPs can be an effective route to forming larger NPs, but such processes are likely to cease once the emerging NPs reach the critical diameter, *d*_c_, for acquiring negative charge under the prevailing local conditions. Once charged, further mass gain by such NPs is only possible via association reactions with the remaining neutral material (*e.g*., with small NPs). The coagulation of negatively charged NPs is very improbable under the prevailing plasma conditions and gas pressures.^66^ The critical diameter *d*_c_ is most dependent on the local electron (*T*_e_) and ion (*T*_i_) temperatures. Under the conditions of present interest, *d*_c_ can be roughly estimated via eq 36, which is derived via the “orbit-limited” charging model and results reported in ref (67)
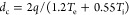
36where *q* is the negative charge on the NP (in units of the electron charge *e*), *T*_e_ and *T*_i_ have units of eV, and *d*_c_ is in nm. For *q* = 1, the minimum *d*_c_ value (∼1.3 nm) is realized in the hot plasma core region **A** (Figure 2d), where *T*_e_ and *T*_i_ are typically ∼1.2 and ∼0.25 eV, respectively. Larger critical diameter values are predicted in region **B** (*d*_c_ ∼ 1.5–3 nm given *T*_e_ ∼ 0.5–1 eV and *T*_i_ ∼ 0.16–0.2 eV) and, particularly, in the colder regions **C** near the cylindrical reactor wall (*d*_c_ ∼ 10 to 13 nm). This implies that particle transfer processes tend to flatten the distribution of NP sizes within the reactor volume. However, as shown below, the Rayleigh scattering cross sections for NPs with such small diameters are too small to make any significant contribution to the measured BA.

The situation will be very different in the slot regions, however. Here, the EM fields are much reduced, and the volumes support a cold decaying plasma with comparably low *T*_e_ ∼ *T*_i_ ∼ *T*_g_ temperatures. For *T*_g_ ∼ *T*_slot_ ∼ 0.025 eV, the upper limit value for growth of neutral NPs is much increased, to *d*_c_ = 46 nm (from eq 36) or without limit in the case of significant plasma decay. This implies that negatively charged small NPs that transport into the slot regions will be neutralized (adjusting to the new local balance of positive ions and electrons) and can associate with each other until they attain much larger *d*_c_ values. As shown below, particles with diameters in the 50–100 nm range will have substantial Rayleigh scattering cross sections.

NP charging could also account for the observed *z*-profile of the BA, which drops steeply at *z* values corresponding to the top and bottom of the slot (Figure 1d). The walls of the aluminum slot are likely to be coated with a thin, negatively charged, insulating oxide layer.^68^ This will serve to repel approaching negatively charged gas phase species and thus reduce the near wall NP concentrations and the probability of them growing and coagulating to form the larger NPs that can be expected to make the dominant contribution to any Rayleigh scattering and/or absorption (see Section 6.2). As noted above, growth and accumulation of larger NPs in the main reactor volume are considered much less likely, given the low critical sizes *d*_c_ for negative charging and the steady gas flow available for transporting emerging clusters/NPs into downstream regions. The optical properties of the NPs will also depend on the (unknown) dielectric function ϵ(λ) of the particle material. We note that the reported optical and electrical properties of a-Si:C:H films grown by plasma enhanced CVD show a range of absorption coefficients^69−71^ and conductivities ranging from dielectric^72^ to conductive.^73^ The possibility of probing ϵ(λ) for the present NPs is considered below.

At a more detailed chemical level, successful clustering (*e.g*., process 35) requires that there be few loss mechanisms for radical sites on the surface of NPs in the cold, H-depleted slot regions. Reactions with H_2_, for example, merit consideration, but we note that the similar radical loss process
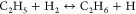
37is mildly endothermic (Δ*H* ∼ 0.15 eV) and heavily suppressed at low *T*_g_ due to the marked decrease in rate coefficient (down to *k*_37_(300 K) ∼ 10^–21^ cm^3^ s^–1^, *cf. k*_37_(3000 K) ∼ 4 × 10^–12^ cm^3^ s^–1^ in the hot plasma core). [The rate coefficients of the respective reverse reactions are *k*_–37_(300 K) ∼ 3.2 × 10^–17^, *cf. k*_–37_(3000 K) ∼ 2.2 × 10^–10^ cm^3^ s^–1^ (ref (74))]. These differences imply that the balance of radical sites in hotter regions of the reactor will be largely determined by the balance between the rates of such loss reactions (with H_2_) and production reactions (H abstraction reactions induced by an incident H atom). The rate coefficients of the clustering processes (*e.g., k*_34_ and *k*_35_) are not known. Reported theoretical and experimental rate coefficients for the aforementioned small radical-molecule association reactions span wide ranges: *T*_g_(400 K) values for *k*(C_2_H_4_ + C_2_H_3_ → C_4_H_7_) and *k*(C_2_H_4_ + C_2_H_5_ → *l*-C_4_H_9_) span the respective ranges ∼3 × 10^–15^ to 2 × 10^–13^ cm^3^ s^1^ and ∼2 × 10^–17^ to ∼2 × 10^–14^ cm^3^ s^–1^ (ref (65)). These values encourage the view that the rate coefficients *k*_34_ and *k*_35_ in the slot regions would be <10^–13^ and <10^–14^ cm^3^ s^–1^, respectively, and that such slow clustering could explain the observed incubation period for BA development (Figure 1).

The (C_3_H_4_)Si(C_3_H_4_) “brick” (and other Si_*z*_C_*y*_H_*x*_ species that might play similar roles) is perceived as key for clustering under the prevailing conditions. The resulting clusters/NPs will survive and accumulate in the stagnation volume within the slots and, progressively, further into the side arms. Neutral particles could also participate in the adsorption/desorption processes at the slot walls. Traditional models of dust particle growth in MW activated C/H/Ar gas mixtures,^75^ lacking such “brick” and (CH_3_)_2_Si=CH_2_ species, offer no such routes to clustering at low *T*_g_. However, pure hydrocarbon clustering processes have been observed in radio frequency and MW activated C_2_H_2_/Ar plasmas at low gas pressures and temperatures (*e.g., p <* 1 Torr and *T*_g_ < 700 K) and high electron temperatures (*e.g., T*_e_ > 2 eV).^76^ Compared with typical diamond deposition regimes involving higher H_2_ fractions and process pressures, such plasma conditions should result in a much higher [C_2_H]/[C_2_H_2_] ratio since C_2_H loss via the H-shifting reaction C_2_H + H_2_ → C_2_H_2_ + H will be greatly reduced. The surviving C_2_H radicals will drive effective polymerization, via C_2_H addition to C_2_H_2_ and other hydrocarbons,^77,78^*e.g.*, to polyynes C_*n*_H_2_, that undergo further molecular growth processes resulting in the nucleation of initial NPs.^76^ This recent study^76^ involved time-resolved laser scattering and extinction diagnostics together with mass, optical emission, and laser absorption spectroscopy methods to explore the dynamics and growth of the NP cloud in a MW activated 50%C_2_H_2_/50%Ar plasma operating at low *p* (0.38 Torr). Characteristic times in the tens to hundreds of second range were identified for NP growth, initially in the plasma region due to C_2_H_2_ dissociation and then for transfer of large (>100 nm) NPs beyond the plasma region. We note that such pure hydrocarbon clustering processes are unlikely to be effective under standard diamond CVD conditions. In the cold off-plasma regions where NPs might grow, the C_2_H concentrations are much too low (Figure 2e). [C_2_H] is high in hot regions, however, but temperatures in the range *T*_g_ ∼ 2000 to 3000 K are unfavorable for NP survival.^75^

## 6 Rationalizing the BA and Its Dependence on Process Conditions

The probed column passes through the hot plasma core, the cooler periphery of the reactor volume, and the side arms that support the cavity defining mirrors. *T*_g_ and the gas phase composition vary greatly along this column. Absorption and scattering could both contribute to the observed loss of probe radiation in the cavity and contribute to the observed delayed growth of Δ*k* at λ ∼427 and ∼656 nm. Here, we consider possible contributions to the probe beam attenuation from absorption by molecular species, then from scattering and absorption by larger species, and their likely relative importances.

### 6.1 Possible Contributions from Small Molecule Absorption

Previous analyses of MW activated dilute Si/C/H gas mixtures identified Si, SiCH_2_, and SiC_2_ as the most stable variants within the respective SiH_*x*_ (*x* = 0–4), SiCH_*x*_ (*x* = 0–3), and SiC_2_H_*x*_ (*x* = 0–2) families.^32^ Of these, only Si=CH_2_ shows (albeit weak) electronic absorption at wavelengths as long as 656 nm.^79^ The absorption spectrum of atomic Si comprises isolated lines, none at the wavelengths of current interest.^80^ Ground state SiH and SiC_2_ species only absorb at wavelengths shorter than, respectively, ∼425^81^ and ∼500 nm,^58^ and the origin of the first electric-dipole allowed transition of Si=CH_2_ is at ∼330 nm.^82^Sections 4 and 5 suggested routes to forming SiC_*y*_H_*x*_ (*y* ≥ 3, *x* = 0 and 1) species and noted that the proposed RRE scheme might favor ejection of species containing multiple Si–C bonds, *e.g.*, nonlinear variants of SiC_*y*_H_*x*_.

Spectroscopic data for SiC_*y*_H_*x*_ (*y* ≥ 3) species are limited. Si(CH_3_)_3_ (eq 21) is a saturated radical. Its longest wavelength absorption lies at UV wavelengths,^83^ as does the lowest energy absorption of closed shell species like 1,1-dimethylsilylene, (CH_3_)_2_Si=CH_2_.^84,85^ Kokkin et al.^22^ reported data for various linear SiC_*y*_H radicals. These all have a doubly occupied σ_Si_ orbital and an unpaired electron in the highest occupied π molecular orbital (a singly occupied (SO) MO). Thus, the linear SiC_*y*_H species support π ← σ_Si_ absorptions in the visible. Theory predicts that these transitions are weak but relatively much stronger for *y* = odd and effectively forbidden for *y* = even. Linear SiC_*y*_H species also show strong π*(LUMO) ← π(SOMO) absorptions at UV wavelengths. The two types of transition show different trends with an increasing *y*. The π* ← π energy separation decreases with increasing *y* (*i.e*., the transition wavelength shifts further into the visible for larger *y*), and it has been estimated that such transitions might fall in the visible region relevant for the DIBs by *y* ≥ 13.^22^ The binding energy of the σ_Si_ orbital is rather insensitive to *y*, however. Thus, the weak π ← σ_Si_ transitions blue-shift with increasing *y*. Electronic spectroscopy data for nonlinear SiC_*y*_H isomers are sparse, but a low-energy cyclic isomer of SiC_3_H (with the heavy atoms arranged similarly to that in *r*-SiC_3_) has been predicted to show similar π ← σ_Si_ and π* ← π absorptions (centered in the visible and in the UV, respectively).^22^ Current knowledge might thus suggest that SiC_*y*_H species with small, odd *y* would contribute (relatively weak) visible absorption and that SiC_*y*_H species with sufficiently large *y* might also contribute much stronger π* ← π absorptions extending into the visible region.

**Figure 6:**
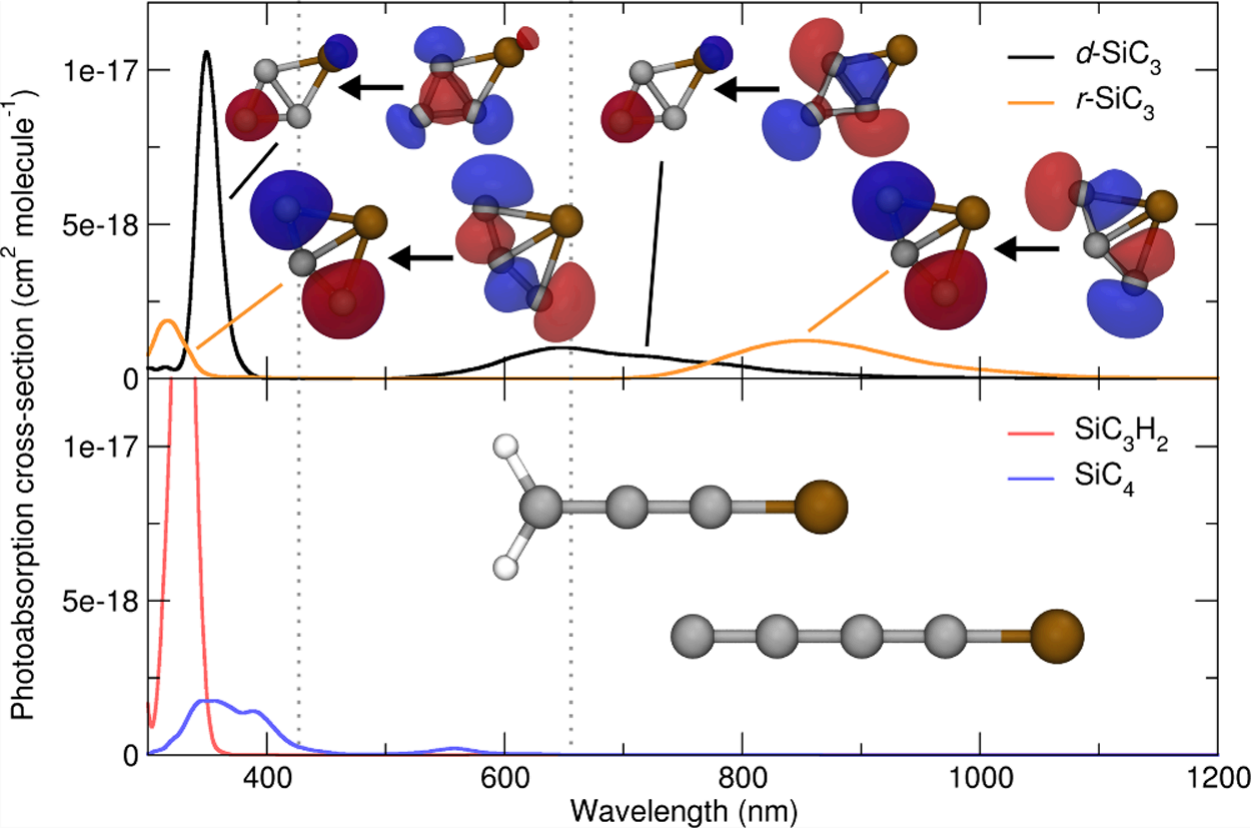
Photoabsorption cross sections for *d*-SiC_3_ and *r*-SiC_3_ (upper panel, black and orange traces, respectively) and *l*-SiC_4_ and SiC_3_H_2_ (lower panel, blue and orange traces, respectively), calculated using the nuclear ensemble approach and EOM-CCSD/cc-pVDZ for the electronic structure. The inset in the upper panel depicts the dominant molecular orbital promotions contributing to each band of *d*-SiC_3_ and *r*-SiC_3_, and the vertical dotted lines show the wavelengths (λ = 427 and 656 nm) at which measurements of the BA signal were made.

Electronic spectroscopy data for SiC_*y*_ and SiC_*y*_H_2_ (*y* ≥ 3) species are also sparse. Figure 6 shows wavelength-dependent photoabsorption cross sections for *r*-SiC_3_ and *d*-SiC_3_, for the lowest energy (linear) isomer of SiC_4_ and for the lowest energy isomer of SiC_3_H_2_, all calculated using the nuclear ensemble approach (NEA)^86^ as implemented in the AtmoSpec code.^87^ All electronic structure calculations were performed using the ORCA program v5.0.4.^88^ To avoid breaking the narrative, details of these calculations are reported in the Supporting Information, along with the calculated vertical excitation energies (VEEs) and oscillator strengths (*f*) for the lower energy transitions of these four species and of *l*-SiC_6_.

Consistent with a passing comment in ref (89), Figure 6 shows that the *d*-SiC_3_ species, with a transannular C–C bond,^49^ exhibits a relatively weak, broad visible absorption attributable to a transition with dominant *b*_1_(LUMO) ← *a*_1_(HOMO) character and a more intense feature at UV wavelengths with dominant *b*_1_(LUMO) ← *a*_1_(HOMO–1) character. The longer wavelength transition has been predicted previously.^90^ Calculations for *r*-SiC_3_ (with a transannular Si–C bond) return broadly similar photoabsorption cross sections, though the low-energy *a*_2_(LUMO) ← *b*_2_(HOMO) band is further red-shifted and the first UV absorption band is (relatively) weaker than that in *d*-SiC_3_. The molecular orbitals involved in these various transitions are shown in the inset of Figure 6. By design, the NEA method does not resolve the excited-state vibrational structure, but the breadth of the predicted long wavelength absorption of both rhomboidal SiC_3_ species implies some change in equilibrium geometry upon electronic excitation and transition energies that fall within the range of many of the visible DIBs.

Rotational spectroscopy studies of SiC_*y*_ (*y* = 5–8) species have shown that the ground states of linear SiC_*y*_ (*y* = even) species have ^1^Σ^+^ term symbols, while those with odd *y* are ^3^Σ^–^ states.^57^ The (higher energy) linear isomer of SiC_3_ (*l*-SiC_3_) has an odd *y* and is thus an open-shell triplet species (and beyond the limits of the level of electronic structure employed in the present work). Linear SiC_4_, *l*-SiC_4_, has a ^1^Σ^+^ ground electronic state and exhibits very weak absorption in the visible (associated with a symmetry forbidden transition) and a more intense band at shorter wavelengths (Figure 6), consistent with previous theoretical results.^90^ The present results for *l*-SiC_6_ (Table S1 in the Supporting Information) confirm previous predictions that the lowest energy transitions in linear SiC_*y*_ species with *y* = 5 and 6 lie in the visible, that they red-shift with increasing *y*, and that they have negligible oscillator strengths.^90^ SiC_*y*_ species with *y* ≥ 9 are predicted to be cyclic, with the Si atom bridging two adjacent ring C atoms,^91,92^ but electronic absorption data for such species are not yet available. SiC_*y*_ “fullerene-like” structures with *y* = 19, 29, 39, 49, and 59 have all been predicted to show absorption at visible wavelengths, but the predicted oscillator strengths are consistently low.^93^ Moving to even larger species, Shi et al.^94^ have calculated spectra for (H-terminated) SiC_*y*_ nanoclusters with *y* = 51, 81, 111, and 201, which are concentrated in the UV but extend across much of the visible region at high *y*.

Returning to Figure 6 (lower panel), the calculated absorption spectrum for the closed shell SiC_3_H_2_ species is also confined to UV wavelengths, as the S_1_ ← S_0_ (*b*_2_(LUMO) ← *b*_1_(HOMO)) transition is electric-dipole forbidden. The key conclusions from this section are that many small SiC_*y*_H_*x*_ species will likely contribute to absorption at ∼427 nm. Fewer will absorb at wavelengths as long as λ ∼ 656 nm and, for those that do, the calculated oscillator strengths are generally low. For larger Si_*z*_C_*y*_H_*x*_ species, however, the tail of the “UV-centered” absorption can be expected to stretch progressively further into the visible with increasing *y* (and *z*).

Given the small predicted absorption cross sections, however, it is hard to envisage how a sufficient column density of SiC_*y*_H_*x*_ species could accumulate to cause the measured attenuation of the probe beam at visible wavelengths. By way of illustration, the analysis of CRDS absorption measurements typically starts with the expression^95^
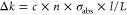
38where σ_abs_ is the absorption cross section, *n* and *l* are, respectively, the number density of species contributing to the BA and the column length over which they are distributed, *L* is the intermirror separation (85 cm), and *c* is the speed of light. Focusing on the λ ∼ 656 nm data under peak BA conditions, Δ*k* (*t* = 300 s) ∼ 1 × 10^6^ s^–1^, from which it follows that *n* × σ_abs_ × *l* ∼ 2.8 × 10^–3^. Assuming σ_abs_ ∼ 10^–18^ cm^2^ (*i.e*., similar to the largest value reported for *d*-SiC_3_ in Figure 6) and *l* = 12 cm (*i.e.*, the internal diameter of the reactor), this would require a species number density of ∼2.3 × 10^14^ cm^–3^. Reference to Figure 5 shows that such a number density is comparable to (and, at most *r*, greater than) that of the most abundant radical (CH_3_) along the probed column and much greater than the estimated total Si concentrations presented at the end of Section 6. Small molecule absorption is thus a wholly unphysical explanation for the observed BA given that only a (small) fraction of all species present will contribute absorption at 656 nm.

### 6.2 Possible Contributions from Scattering

Section 5.2 outlined a scenario where small SiC_*y*_H_*x*_ species formed by RRE of the quartz window could be processed while being transported to and ultimately accumulating in the cool slot regions between the main reactor volume and the side arms, where they can seed the growth of larger NPs. The emerging particles may absorb (probably only weakly at visible wavelengths, given available data for amorphous Si:C:H films^96,97^) but can also scatter the probe laser radiation. Here, we show that a scattering-dominated explanation based on RRE of the quartz window and Si-nucleated NP growth in the slot regions along the probed column can accommodate the shape and magnitude of the experimentally observed Δ*k*/d*t* versus *X*_0_(CH_4_) dependences at both probe wavelengths and the observed spatial localization.

#### 6.2.1 Estimating Reactive Radical Etching Rates

Building on Section 4, we first derive an expression for the RRE rate as a function of [H] and [C_*y*_H_*x*_] just below the quartz window and the internal window surface temperature, *T*_SiO2_. For a H_2_ plasma, with negligible etching of the SiO_2_ surface, we can anticipate the following balance between the rates of destroying and restoring Si–O–Si bridges
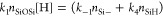
39where the various *n* define the concentrations of surface sites.

Simultaneously, the balance between the Si– and SiH sites will be largely determined by the much more rapid interconversions described in reactions 2 and 3
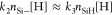
40

Equations 39 and 40 allow estimation of the ratio of broken versus surviving Si–O–Si bridges, *i.e.*,
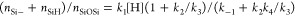
41

Given [H] < 3.5 × 10^15^ cm^–3^ and *T*_SiO2_ ∼ 900 K (at *p* = 75 Torr, Figure 4a) and the reaction barriers from ref (8), this ratio will be ≪1, consistent with the negligible SiO_2_ etching rates observed experimentally.

Upon CH_4_ addition, the passivation processes (eq 6) will compete with the Si–O–Si bond restoration processes, reducing their rate by a factor
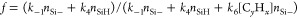
42

These rival C_*y*_H_*x*_ addition processes promote eventual SiO_2_ etching. The remaining (1 – *f*) contribution from eq 42 is assumed to represent the CH_4_-enabled etching rate
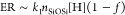
43which when combined with eqs 40 and 41 gives the following functional dependence for the ER:
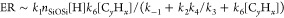
44

The rate coefficients in eq 44 are not well established, and some will be surface condition-dependent, *e.g., k*_1_ (and the ER) may well be larger for a roughened surface. Nonetheless, eq 44 with the calculated [H] and [C_*y*_H_*x*_] concentrations (from Figure 4) and the following illustrative set of rate coefficients: *k*_1_ = 10^–10^exp(−10444/*T*_SiO2_) cm^3^ s^–1^, *k*_–1_ = 10^14^exp(−19730/*T*_SiO2_) s^–1^, and *k*_4_ = 10^14^exp(−22050/*T*_SiO2_) s^–1^ (where we have adopted reaction barriers reported in ref (8)), together with *k*_2_ = 2 × 10^–10^ cm^3^ s^–1^, *k*_3_ = 2 × 10^–11^ cm^3^ s^–1^, and *k*_6_[C_*y*_H_*x*_] = 10^–10^ × ([CH_3_] + [C_2_H_3_]),^98^ [with *k*_6_ in cm^3^ s^–1^], returns a nonmonotonous ER(*X*_0_(CH_4_)) dependence, with a broad maximum at *X*_0_(CH_4_) ∼ 0.9 to 1.8% and ER ∼ 3 nm min^–1^ at the hottest central region (*r* < 10 mm). Qualitatively, at least, the ER(*X*_0_(CH_4_)) dependence described here (plus the assumption that NPs accumulate and grow in the slot regions) can accommodate the experimentally observed variation of Δ*k*(*t*) with *X*_0_(CH_4_).

#### 6.2.2 Time Dependence of the BA

OES studies show that the plasma parameters re-establish within 1–2 s of introducing CH_4_ to a pre-existing H_2_ plasma.^25^ The reactor volume, taken together with the chosen flow rate (*F*_total_ = 565 sccm) and pressure (*e.g., p* = 75 Torr), ensures that the turn over time for the gas in the main body of the reactor is of order ∼10 s. Many factors contribute to the >100 s induction time before the appearance of detectable BA following the introduction of CH_4_ and the subsequent Δ*k*(*t*) dependence. These include the time to initiate etching of the quartz surface, the time taken to transport the etched species and the plasma processed SiC_*y*_H_*x*_ clusters/NPs to the slot regions, the time these species then spend growing to NPs with appreciable absorption and/or scattering cross sections, and the time-evolving column length along which they are distributed. The development of the BA could also contain elements related to the initial fast deposition of organosilicon species on the (metal) reactor walls. With increasing coverage of the wall surface, loss of SiC_*y*_H_*x*_ species (by deposition) will be progressively mitigated by production from competitive etching and desorption processes—leading to a rise in the total amount of SiC_*y*_H_*x*_ material in the entire reactor (including the slot regions). At early times after CH_4_ addition, SiC_*y*_H_*x*_ material (*e.g.*, clusters derived from the “bricks”) will be accumulating in the slot regions, small NPs will be forming, and existing NPs will be growing larger. SiC_*y*_H_*x*_ material entering the slot regions will be accommodated (with increasing efficiency) by addition/chemisorption reactions on the surface of larger NPs that can maintain neutrality up to much larger *d* < *d*_c_ ∼ 50 nm, as described in Section 5.2. As shown below, the scattering cross sections, σ_sc_, scale as ∼ *d*^6^ and scattering by NPs with *d*_c_ ∼ 50 nm (or slightly larger) could account for the measured Δ*k*(*t*) dependences.

The later time parts of the various Δ*k*(*t*) traces in Figure 1 all show near-quadratic increases, *i.e*.,
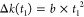
45where *t*_1_ = *t – t*_ind_ and the *b* coefficients [with units of s^–3^] and induction times *t*_ind_ [s] are process condition-dependent. By way of illustration, the *F*(CH_4_) = 5, 15, 25, 30, and 35 sccm data in Figure 1b (all for *p* = 75 Torr and probing at λ = 427 nm) are well described by, respectively, *b* = 26, 95, 30, 15.5, and 4.3 s^–3^ and *t*_ind_ = 195, 120, 135, 200, and 250 s. Similarly, the *F*(CH_4_) = 15 sccm and *p* = 100 and 125 Torr data in Figure 1a are reproduced well by, respectively, *b* = 26 and 4.3 s^–3^ and *t*_ind_ = 167 and 350 s. The qualities of these various fits are shown by the (appropriately color-coded) dashed lines in Figure 1a−c. However, we reiterate that these simple functions mask a multitude of complexity: for any local conditions and moment in time, the NPs will have a range of sizes and masses, the distributions of which will surely vary along the probed column.

#### 6.2.3 Modeling the Dynamics of Larger NPs in the Slot Regions

The largest NPs will be dominant contributors to any scattering. Here, we seek to trace the dynamics of such NPs in the slot regions. The NPs are treated as monosized, porous spheres with mass *m*(*t*_1_) and volume *V*(*t*_1_) = *m*(*t*_1_)/ρ, and we assume a broadly constant (*i.e*., time-independent) density ρ and concentration [NP]. This latter assumption implies a growth regime wherein *m*(*t*_1_) increases near-linearly; the incoming (and existing) SiC_*y*_H_*x*_ material is assumed to accommodate preferentially on pre-existing NPs, rather than nucleate new NPs. An alternative limiting regime, where incoming smaller particles preferentially associate with each other, should manifest as increasing [NP](*t*_1_). A linear growth of [NP](*t*_1_) (and thus of the column density {NP}(*t*_1_)) would be reflected in a linear increase in Δ*k* since Δ*k*(*t*_1_) ∼ {NP}). The experimentally observed near-quadratic Δ*k* vs *t*_1_ dependence (Figure 1 and eq 45) is consistent with growth of *m* and *V* (and thus of the effective scattering cross section σ_sc_) at roughly constant average [NP]. Thus, the illustrative model is based on the preferential growth of the larger NPs in the slot region.

Equation 46 is the simple balance equation for the time-evolving NP volume *V*(*t*_1_):
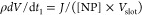
46where *J* [g s^–1^] is the mass of SiC_*y*_H_*x*_ material accommodated per second on all NPs in the slot region, balanced by the same amount of SiC_*y*_H_*x*_ material entering the slot region (the cross-sectional area of which is *S*_slot_ = 2.3 cm^2^). The volume of each slot is *V*_slot_ = 5.15 cm^3^. Under quasi-steady-state conditions (constant *J*), the NP volume *V* will increase linearly with time, *i.e.,*
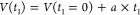
47where the premultiplier *a* = *J* /([NP] × *V*_slot_ × ρ) is a constant factor for a given set of process conditions and *V*(*t*_1_ = 0) ≪ *V*(*t*_1_ > 100 s). The scattering cross section of the NP will show a near-quadratic time dependence due to the σ_sc_ ∼ *d*^6^ functional dependence:
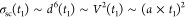
48

To develop this picture further, we seek possible sets of parameters that (i) succeed in reproducing the observed dynamics of the BA, *i.e*.,
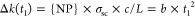
49(where the NP column density {NP} = [NP] × *l*_slot_, the total length of the two slot regions *l*_slot_ = 4.5 cm, and *c* and *L* are as in eq 38), and (ii) accord with the Rayleigh formula for the scattering cross section, σ_sc_.
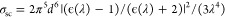
50

Equations 46 through 50 show that the factor *b* (determined from Figure 1a–c and given above) obeys the approximate proportionality *b* ∼ [NP] × *a*^2^/λ^4^. Thus, for any chosen (unknown) [NP], we can estimate a factor of *a* ∼ *V*(*t*_1_)/*t*_1_ and all other parameters of the growing NP (including a complex dielectric function ϵ(λ) = ϵ_1_(λ) + *i*ϵ_2_(λ)) that succeeds in reproducing the BA dynamics observed under the particular process conditions.

This illustrative analysis assumes negligible NP absorption (σ_abs_ ≪ σ_sc_) at λ = 427 nm. The relative contribution that the absorption cross section, σ_abs_, makes to the total cross section (σ_abs_ + σ_sc_) increases with λ, and (weak) absorption might account for the (modest) deviation (Figure 1c) from the predicted Δ*k* ∼ λ^–4^ dependence (eq 50). As elsewhere,^99^ the wavelength dependence of the absorption cross section was estimated via
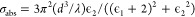
51assuming an ϵ_2_(λ) ∼ λ^3^ dependence and that ϵ_2_(λ) ≪ ϵ_1_(λ) at visible and near UV wavelengths.^100^ Several further assumptions are required to determine the value for ϵ_2_(λ). The NPs are assumed to be spherical and porous. Racine et al.^101^ have reported refractive index *n* and density ρ data for a-Si:C:H films, finding *n* = 2.3 (at λ = 633 nm) for a wide range of Si:C ratios, implying that ϵ_1_ ∼ 5.3 for an assumed nonporous film with ϵ_2_(λ) ≪ ϵ_1_(λ). Berryman^102^ reported ∼36% porosity for randomly close packed monosized solid spheres. The illustrative calculations reported here assume NPs with ∼45 ± 5% porosity, a constant ϵ_1_ ≈ 3, and ρ = 1.05 g cm^–3^ (given the value ρ ≈ 2.1 g cm^–3^ measured for nonporous 20%Si:C films^101^). Given these fixed values of ϵ_1_ and ρ, the experimental results in Figure 1a–c can all be accommodated within the framework of this model and eqs 46–51 by adopting a function ϵ_2_(λ) = 0.008 × (λ/427).^3^ Note that the present study is silent regarding the stoichiometry of the larger NPs. One limiting model might assume NP growth by assembly of many elementary (C_3_H_4_)_2_Si “bricks”. Such NPs would have a heavy atom (C:Si) ratio ∼6 and an H atom content H:Si < 8. Other models might assign a much greater role for C_*y*_H_*x*_ species addition, as per eqs 28 and 32, yielding NPs with higher C:Si ratios.

### 6.3 Comparisons with the Experiment

Table 2 reports parameters determined for two sets of process conditions used when recording the data shown in Figure 1. The first three data columns in Table 2 are for “peak” BA conditions (*p* = 75 Torr, *F*(CH_4_) = 15 sccm) and assume three different average NP concentrations in the slot regions: [NP] = 10^8^, 10^9^, and 10^10^ cm^–3^. The final two columns should be viewed in tandem with the second data column. All three assume [NP] = 10^9^ cm^–3^ and *p* = 75 Torr but different *F*(CH_4_) values. The time-dependent parameters reported in Table 2 are for *t*_1_ = 130 s, *i.e.,* at the end of the steeply rising Δ*k* trace measured under peak BA conditions, for which Δ*k*(*t*_1_ = 130 s; λ = 427 nm) = 1.6 × 10^6^ s^–1^ (Figure 1c). Recalling Figure 1a,b, the very similar Δ*k*(*t*_1_) dependences observed for *p* = 75 Torr, *F*(CH_4_) = 35 sccm and for *p* = 125 Torr, *F*(CH_4_) = 15 sccm imply that both can be modeled by the same *b* value (4.3 s^–3^ via eq 49). Within the prevailing approximations, therefore, the final data column in Table 2 should also be appropriate for the *p* = 125 Torr, *F*(CH_4_) = 15 sccm conditions.

**Table 2:** Illustrative Sets of Parameters for Different Assumed NP Concentrations, [NP], and the Mass Accommodation Rates, *J*, of SiC_*y*_H_*x*_ Material Deposited on the Total Surface Area, *S*, of All NPs in One Slot Region, for MW Discharge Regimes Involving Various *F*(CH_4_), All for *p* = 75 Torr, Estimated at *t*_1_ = 130 s^a^

*F*(CH_4_)/sccm	15	15	15	30	35
[NP]/cm^–3^	1 × 10^8^	1 × 10^9^	1 × 10^10^	1 × 10^9^	1 × 10^9^
*d*/nm	99	68	46	50	40
σ_sc_(427 nm)/ cm^2^	9.3 × 10^–12^	9.3 × 10^–13^	9.3 × 10^–14^	1.5 × 10^–13^	4.1 × 10^–14^
σ_abs_(427 nm)/ cm^2^	2.1 × 10^–13^	6.8 × 10^–14^	2.1 × 10^–14^	2.8 × 10^–14^	1.4 × 10^–14^
σ_sc_(656 nm)/ cm^2^	1.7 × 10^–12^	1.7 × 10^–13^	1.7 × 10^–14^	2.7 × 10^–14^	7.4 × 10^–15^
σ_abs_(656 nm)/ cm^2^	5.1 × 10^–13^	1.6 × 10^–13^	5.1 × 10^–14^	6.5 × 10^–14^	3.4 × 10^–14^
*J*/g s^–1^	2.1 × 10^–9^	6.7 × 10^–9^	2.1 × 10^–8^	2.7 × 10^–9^	1.4 × 10^–9^
*S*/cm^2^	0.16	0.74	3.4	0.40	0.26
ER/nm min^–1^	1.1	3.6	11	1.4	0.8

a The final row reports the average material etching rate, ER, from the quartz window, estimated, assuming that etching occurs only from the hottest central (*r* < 3 cm) region.

Several aspects of the data in Table 2 merit note. The σ_sc_ and σ_abs_ values are mostly within the range of applicability of the Rayleigh formula (*d* < 0.2 × λ).^99^ Absorption is predicted to make a relatively minor (<25%) contribution to the total cross section (σ_abs_ + σ_sc_) at λ = 427 nm, but its relative contribution is larger at λ = 656 nm. Absorption is predicted to be the dominant contributor to σ_tot_ for NPs with *d* < 65 nm at this longer wavelength. At both wavelengths, the relative contribution from σ_abs_ is predicted to decrease with increasing *d*. Second, the (large) total surface areas *S* = π*d*^2^ × [NP] × 2×*V*_slot_ of the larger NPs means that they are likely to serve as efficient sinks for SiC_*y*_H_*x*_ species, clusters, and smaller NPs. As noted below, this should lead to some redistribution of the diffusional fluxes of such species within the reactor volume in favor of the slot regions. Third, the current modeling reproduces the observed decreases in the rates of NP formation (and quartz window etching) with increasing *F*(CH_4_). Recalling that the Δ*k*(*t*_1_) dependences measured for *p* = 75 Torr, *F*(CH_4_) = 35 sccm and for *p* = 125 Torr, *F*(CH_4_) = 15 sccm imply essentially identical *b* values, the data in columns 2 and 5 of Table 2 also reproduce the observed decrease in the NP formation and quartz window etch rates with increasing *p*.

A crude estimate of the ER of the quartz window (averaged over the main etching area, *S*_hot_ = π(*r*/2),^2^ which is here taken as the hot central region of the window with *r* = *R*/2) can be gained using *J* and the following picture. The organosilicon material etched from the central region of the window is pictured expanding in the radial and axial downstream directions by diffusion and gas flow processes. Upon reaching the wall of the cylindrical reactor (*i.e., r* = *R*), further transfer of Si-containing material into the downstream region will be mainly axial. In this quasi-steady-state regime, the integral axial flux of silicon will be constant and broadly match the initial flux of the etched material. We note that the main gas flow under steady state conditions, *i.e.*, the total axial mass transfer ∫2π*r*ρ_gas_*v*_*z*_d*r* ≈ *J*_0_ [g s^–1^] (where ρ_gas_, *v*_*z*_, and *J*_0_ are the total local gas density, the axial velocity, and the supplied mass rate, respectively), is inevitably preserved in the radial reactor cross sections at lower *z* if etching/deposition processes at the side walls are negligible. The net near-zero radial velocities should also prevail in the lateral stagnation flow regions, including the slots and bellows, and provide the residence times necessary for NP growth.

During those periods when the BA is increasing, however, there is likely to be some net (but still unestablished) radial diffusional transfer of organosilicon material (*i.e.*, “bricks”, small NPs, etc.) into the slot regions to drive the growth of larger NPs. The derived mass flux *J*/*S*_slot_ [g cm^–2^ s^–1^)] of SiC_*y*_H_*x*_ material into the slot regions, along with an assumption of the C/Si ratio in the material (∼6), allows estimation of the silicon flux into the slots and the silicon concentration near the slots, which we take as a crude estimate of the asymptotic downstream axial flux. Recognizing the respective cross-sectional areas of the reactor (*S*_r_ = π*R*^2^), *S*_slot_ and *S*_hot_, we can then derive an estimate of the flux of etched silicon and thus of the ER of the quartz window: ER × *S*_hot_ ∼ *J × S*_*r*_/(*S*_slot_ × *m*_brick_), where *m*_brick_ ∼ 108, the mass of the C_3_H_4_SiC_3_H_4_ “brick” in atomic units. Given a quartz window density ρ_SiO2_ = 2.3 g cm^–3^, this approach yields the average ER values (in nm min^–1^) shown in Table 2. Similar ER values can also be deduced assuming the following balance in the slot regions—that the volume averaged rate of Si supply (∼ER × *S*_hot_)/*V*_reactor_ (where *V*_reactor_ is the reactor volume) is equal to the rate at which Si material is consumed in NP growth. It is instructive to compare these deduced ER estimates with the quartz etching rates (ER_H2_ ∼ 1 to 200 nm min^–1^) measured using a MW activated H_2_ plasma at quartz temperatures *T*_SiO2_ in the range of 1063–1573 K.^6^ Extrapolating the data to the temperature of current interest yields ER_H2_(*T*_SiO2_ = 900 K) ∼ 0.1 nm min^–1^. Notwithstanding that the H atom fluxes in the MW activated H_2_ plasma were probably higher than those in the present work, the ER_H2_(900 K) value extrapolated from ref (6) is significantly lower than the RRE rate estimates in Table 2, reinforcing the view that H atoms and C_*y*_H_*x*_ radicals have a synergistic role in the RRE of quartz.

As noted above, contamination of the plasma (and thus the as-grown diamond) is a problem with most CVD reactors. The foregoing estimates of the total supply of etched silicon atoms and the previous results from our 2D model calculations of SiH_4_/CH_4_/H_2_ mixtures with different (known) flow rates of SiH_4_ source gas^32^ allow the following crude estimates of the total Si concentration close above the substrate in the present regimes: ∼7 × 10^12^, ∼2.5 × 10^12^, and ∼10^11^ cm^–3^ for *p* = 75, 125, and 150 Torr, respectively, for *F*(CH_4_) = 15 sccm and *P* = 1.4 kW in all cases.

## 7 Conclusions

The present analysis demonstrates that the BA observed in CRDS studies of MW activated CH_4_/H_2_/(Ar) gas mixtures operating at low *p* and specific *F*(CH_4_) values can be attributed to NPs that accumulate and grow in the slot regions on opposing sides of the reactor. The illustrative modeling succeeds in explaining the form of the observed Δ*k*(*t*) dependences, which are deduced to involve contributions from both scattering and absorption, and their variations with probe wavelength. The observed *F*(CH_4_) and *p* dependences (Figure 1) reflect the requirements for RRE of the quartz window: high *T*_SiO2_ together with suitably high near-window H atom and C_*y*_H_*x*_ radical concentrations. The measured spatial localization (Figure 1d) reflects the slot geometry. The steep decreases in Δ*k* at both high and low *z* can be attributed to the displacement of larger (and negatively charged) NPs from the sheath near the aluminum walls that are expected to be coated with a negatively charged insulating oxide layer and may be further exacerbated by the drop in radical concentrations near the walls (that will reduce the rates of the association reactions that underpin NP growth).

The present study is by no means the first proposing NP formation in MW activated CH_4_/H_2_ gas mixtures, but its design—with accessible stagnation regions adjacent to the main reactor volume—does offer unusually favorable opportunities for observing such NPs. Homogeneous nucleation of nanodiamond particles in a MW plasma was proposed as long ago as 1989^103^ and has since been variously proposed to account for features of the optical emission accompanying diamond growing plasmas.^75,104^ We note that quartz (in the form of the reactor tube,^103^ as a bell jar surrounding the plasma volume,^75^ or as the entrance window for the MW radiation^104^) was present in all of these studies but not recognized as a potential promotor of gas phase nucleation. A more recent study of nanodiamond film formation in a MW activated, very low pressure (*p* = 40 Torr), 5% CH_4_ in the H_2_ gas mixture (*P* = 2.5 kW) also calls for a reassessment of the importance of gas phase nanodiamond nucleation under such conditions.^105^ We note that the reactor employed in that study also contained a quartz bell jar and that the pinhole design implemented to prevent the plasma from reaching the Si substrate surface (covered by a Mo disk with a pinhole, 4 mm in height and diameter) might constitute a stagnation volume capable of supporting unrecognized Si-enhanced nucleation at or near the base of the pinhole. However, we also note that, just as in the present work, the pressures at which NP formation was observed are significantly lower than those used in most contemporary diamond CVD reactors and that the reactor design afforded an under-pumped stagnation region that could facilitate NP growth. The present study shows that silicon-containing components can enter the gas phase via the RRE of any suitably hot quartz (and Si) surfaces within the reactor. Similar source materials (the quartz components exposed to the plasma and the Si substrate) were distinguished (and decoupled) in a recent study of the distribution of SiV centers in polycrystalline diamond films grown by MW CVD using CH_4_/H_2_ gas mixtures.^12^ The present study also reinforces the view that Si atoms can play an important role in stabilizing small carbon clusters, as seen also in the case of amorphous and crystalline silicon carbide nanoclusters deposited on a cooled substrate following pyrolysis of tetramethylsilane in a hot wall reactor at a range of temperatures (1000–1300 °C) and pressures (8–20 mbar),^106^ and as has also been suggested in a recent study of successful growth diamond in liquid metal.^23^ However, it adds little to the continuing debate about the importance (or not) of homogeneous gas phase nucleation of small nanodiamond seed particles in MW activated C/H/(Ar) gas mixtures operating at the higher *p* and *P* values used in most commercial diamond CVD.

It is instructive to compare the gas phase RRE mechanism proposed here with the much more widely established RIE mechanism. Surface bonds are broken by energetic ions (in RIE) rather than H atoms (in RRE), and the passivation of unsaturated bonds is typically achieved by species (*e.g.*, fluorocarbons, hydrofluorocarbons, *etc*.) ejected from a polymeric film formed above the etched material (in RIE)^2^ rather than incident C_*y*_H_*x*_ radicals (as in the present RRE case). For the specific case of F atoms interacting with SiO_2_, a multistep radical mechanism wherein F atoms participate in both Si–O bond breaking and Si– site passivation has been proposed previously.^107^ The RRE mechanism provides an opportunity both for ion-free etching and decoration (functionalization) of the SiO_2_ surface by hydrocarbon C_*y*_H_*x*_ species (*e.g.*, CH_3_ and C_2_H_3_), but it does require elevated temperatures, *e.g., T*_SiO2_ > 400 °C. As such, RRE might offer interesting opportunities in the fields of area-selective atomic layer deposition (ALD)^108^ and in the reformation of SiCH_3_ groups in damaged SiOCH low-*k* dielectrics by use of the appropriate H atom and CH_3_ radical flux ratios.

We anticipate similarities in the interactions of H atoms and C_*y*_H_*x*_ radicals with hot SiO_2_ and Si_3_N_4_ surfaces^109^ and propose that an RRE mechanism similar to that advanced in the present work may contribute to the observed etching of the Si_3_N_4_ surface when Si_3_N_4_/GaN substrates are exposed to MW plasma-activated CH_4_/H_2_ gas mixtures.^110^ Under conditions broadly similar to those used in the present work, Hao et al. showed Si–C bond formation in a thin interfacial layer between the as-grown diamond and the Si_3_N_4_/GaN substrate and a reduction in the thickness of the Si_3_N_4_ dielectric layer (from 10 to 2 nm).^110^

Finally, we note that the proposed RRE mechanism also has potential implications for many studies of diamond CVD on silicon substrates. Most such substrates will initially be covered with a thin SiO_2_ layer. The substrate temperature will typically be higher than the maximum *T*_SiO2_ values considered here, and the high [H] and [C_*y*_H_*x*_] densities near the substrate^18^ (*e.g.,* [H](*z* = 0) ≈10^15^ cm^–3^, [CH_3_](*z* = 0) ≈8.5 × 10^13^ cm^–3^ , and [C_2_H_3_](*z* = 0) ≈10^13^ cm^–3^ just above the substrate center for peak BA conditions) will likely suffice to ensure rapid removal of the SiO_2_ coating by RRE. Thereafter, it is reasonable to anticipate some broadly analogous RRE of the exposed silicon. Incident H atoms etch bare Si, even at room temperature,^111^ and Si etching by a MW activated H_2_ plasma has been inferred by observation of optical emission from Si* and SiH* species at *p* and *P* values appropriate for diamond CVD.^32,112^ Such emissions disappeared promptly upon introducing *F*(CH_4_) = 1 sccm,^32,112^ however, consistent with the change in etched products from SiH_*x*_ species in MW activated H_2_ (and Ar/H_2_) plasmas to SiC_*y*_H_*x*_ species in CH_4_/H_2_ plasmas as proposed in the present RRE model. Lin et al. also demonstrated that etching of a (hot but remote) silicon substrate could act as a source for incorporating Si (and SiV^–^ defects) in a homoepitaxial diamond grown on a pre-existing diamond seed crystal using MW activated CH_4_/H_2_ gas mixtures.^112^ In the more typical situation where a piece of silicon wafer constitutes the substrate, any RRE of the substrate surface will occur in competition with carburization and is likely to be progressively suppressed by the developing thin silicon carbide layer and its subsequent overgrowth by polycrystalline diamond.

## Supporting Information Available

Summary of methods used to (i) optimize geometries and normal-mode frequencies of ground state SiC_*y*_H_*x*_ species featured in Figure 6, (ii) calculate vertical excitation energies (VEEs) to the first few excited singlet (S_*x*_) states, and (iii) simulate the respective UV-visible absorption cross sections. Table showing calculated VEEs and oscillator strengths (*f*) for transitions to the first and selected higher excited states of *d*-SiC_3_, *r*-SiC_3_, *l*-SiC_4_, *l*-SiC_6_, and SiC_3_H_2_ (PDF)

## References

[ref1] Coburn J. W.; Winters H. F. Plasma Etching—A Discussion of Mechanisms. J. Vac. Sci. Technol. 1979, 16, 391–403. 10.1116/1.569958.

[ref2] Donnelly V. M.; Kornblit A. Plasma Etching: Yesterday, Today, and Tomorrow. J. Vac. Sci. Technol., A 2013, 31, 050825 10.1116/1.4819316.

[ref3] Mehedi H.-A.; Ferrah D.; Dubois J.; Petit-Etienne C.; Okuno H.; Bouchiat V.; Renault O.; Cunge G. High Density H_2_ and He Plasmas: Can they be Used to Treat Graphene?. J. Appl. Phys. 2018, 124, 125304 10.1063/1.5043605.

[ref4] Zhou Y.; Li H.; Jung J. E.; Nam S. K.; Donnelly V. M. Effects of N_2_ and O_2_ Plasma Treatments of Quartz Surfaces Exposed to H_2_ Plasmas. J. Vac. Sci. Technol., A 2022, 40, 053002 10.1116/6.0001896.

[ref5] Yurov V. Y.; Bolshakov A. P.; Altakhov A. S.; Fedorova I. A.; Zavedeev E. V.; Popovich A. F.; Ralchenko V. G. Hydrogen Microwave Plasma Etching of Silicon Dioxide at High Temperatures with in situ Low-Coherence Interferometry Control. Vacuum 2022, 199, 110939 10.1016/j.vacuum.2022.110939.

[ref6] Yurov V.Yu.; Bolshakov A. P.; Fedorova I. A.; Popovich A. F.; Zyablyuk K. N.; Altakhov A. S.; Sovyk D. N.; Pivovarov P. A.; Volkov P. V.; Ralchenko V. G. Control of Silicon Dioxide Etching Rate in Hydrogen Microwave Plasma by Addition of Oxygen. Appl. Surf. Sci. 2023, 612, 155834 10.1016/j.apsusc.2022.155834.

[ref7] Peña O.; Muhl S.; López W.; Rodríguez-Fernández L.; Ruvalcaba-Sil J. L. Hydrogen Plasma Etching of Silicon Dioxide in a Hollow Cathode System. Thin Solid Films 2010, 518, 3156–3159. 10.1016/j.tsf.2009.08.042.

[ref8] El-Sayed A.-M.; Watkins M. B.; Grasser T.; Afanas’ev V. V.; Shluger A. L. Hydrogen-Induced Rupture of Strained Si—O Bonds in Amorphous Silicon Dioxide. Phys. Rev. Lett. 2015, 114, 115503 10.1103/PhysRevLett.114.115503. 25839289

[ref9] Shoeb J.; Wang M. M.; Kushner M. J. Damage by Radicals and Photons During Plasma Cleaning of Porous Low-*k* SiOCH. I. Ar/O_2_ and He/H_2_ Plasmas. J. Vac. Sci. Technol., A 2012, 30, 041303 10.1116/1.4718444.

[ref10] Sakaguchi I.; Nishitani-Gamo M.; Loh K. P.; Haneda H.; Hishita S.; Ando T. Silicon Incorporation into Chemical Vapor Deposited Diamond: A Role of Oxygen. Appl. Phys. Lett. 1997, 71, 629–631. 10.1063/1.119812.

[ref11] Ma J.; Richley J. C.; Ashfold M. N. R.; Mankelevich Y. A. Probing the Plasma Chemistry in a Microwave Reactor used for Diamond Chemical Vapor Deposition by Cavity Ring Down Spectroscopy. J. Appl. Phys. 2008, 104, 103305 10.1063/1.3021095.

[ref12] Raj R.; Pradeep K. G.; Rao M. S. R. Formation and Properties of Silicon Vacancies in MPCVD-Grown Polycrystalline Diamond. Bull. Mater. Sci. 2024, 47, 228 10.1007/s12034-024-03284-3.

[ref13] Yang K.; Teng Y.; Zhao W. K.; Tang K.; Fan K. K.; Duan J. J.; Huang Y. M.; Ye J. D.; Zhang R.; Zhu S. M.; et al. Formation Mechanism of SiV in Diamond from Unintentional Silicon Doping by Microwave Plasma Chemical Vapor Deposition. Vacuum 2024, 222, 113027 10.1016/j.vacuum.2024.113027.

[ref14] Hassouni K.; Grotjohn T. A.; Gicquel A. Self-Consistent Microwave Field and Plasma Discharge Simulations for a Moderate Pressure Hydrogen Discharge Reactor. J. Appl. Phys. 1999, 86, 134–151. 10.1063/1.370710.

[ref15] Gu Y. J.; Lu J.; Grotjohn T.; Schuelke T.; Asmussen J. Microwave Plasma Reactor Design for High Pressure and High Power Density Diamond Synthesis. Diam. Rel. Mater. 2012, 24, 210–214. 10.1016/j.diamond.2012.01.026.

[ref16] Bogdanov S. A.; Gorbachev A. M.; Vikharev A. L.; Radishev D. B.; Lobaev M. A. Study of Microwave Discharge at High Power Density Conditions in Diamond Chemical Vapor Deposition Reactor by Optical Emission Spectroscopy. Diam. Rel. Mater. 2019, 97, 107407 10.1016/j.diamond.2019.04.030.

[ref17] Ma J. Exploration of the Gas Phase Chemistry in Microwave Activated Plasmas used for Diamond Chemical Vapour Deposition, Ph.D. Thesis., University of Bristol: 2008.

[ref18] Ashfold M. N. R.; Mankelevich Yu.A. Two-Dimensional Modeling of Diamond Growth by Microwave Plasma Activated Chemical Vapor Deposition: Effects of Pressure, Absorbed Power and the Beneficial Role of Nitrogen on Diamond Growth. Diam. Rel. Mater. 2023, 137, 110097 10.1016/j.diamond.2023.110097.

[ref19] Herbig G. H. The Diffuse Interstellar Bands. Annu. Rev. Astron. Astrophys. 1995, 33, 19–73. 10.1146/annurev.aa.33.090195.000315.

[ref20] MacKay D. D. S.; Charnley S. B. The Silicon Chemistry of IRC+10°216. Mon. Not. R. Astron. Soc. 1999, 302, 793–800. 10.1046/j.1365-8711.1999.02175.x.

[ref21] Sarre P. J.; Hurst M. E.; Lloyd Evans T. SiC_2_ in Carbon Stars: Merrill-Sanford Absorption Bands between 4100 and 5500 Å. Mon. Not. R. Astron. Soc. 2000, 319, 103–110. 10.1046/j.1365-8711.2000.03818.x.

[ref22] Kokkin D. L.; Reilly N. J.; Fortenberry R. C.; Crawford T. D.; McCarthy M. C. Optical Spectra of the Silicon-Terminated Carbon Chain Radicals SiC_*n*_H (*n* = 3,4,5). J. Chem. Phys. 2014, 141, 044310 10.1063/1.4883521. 25084913

[ref23] Gong Y.; Luo D.; Choe M.; Kim Y.; Ram B.; Zafari M.; Seong W. K.; Bakharev P.; Wang M.; Park I. K.; et al. Growth of Diamond in Liquid Metal at 1 atm Pressure. Nature 2024, 629, 348–356. 10.1038/s41586-024-07339-7. 38658760

[ref24] Mankelevich Y. A.; Ashfold M. N. R.; Ma J. Plasma-Chemical Processes in Microwave Plasma Enhanced Chemical Vapour Deposition Reactors Operating with C/H/Ar Gas Mixtures. J. Appl. Phys. 2008, 104, 113304 10.1063/1.3035850.

[ref25] Ma J.; Ashfold M. N. R.; Mankelevich Y. A. Validating Optical Emission Spectroscopy as a Diagnostic of Microwave Activated CH_4_/Ar/H_2_ Plasmas used for Diamond Chemical Vapor Deposition. J. Appl. Phys. 2009, 105, 043302 10.1063/1.3078032.

[ref26] Mahoney E. J. D.; Truscott B. S.; Mushtaq S.; Ashfold M. N. R.; Mankelevich Yu.A. Spatially Resolved Optical Emission and Modeling Studies of Microwave-Activated Hydrogen Plasmas Operating under Conditions Relevant for Diamond Chemical Vapour Deposition. J. Phys. Chem. A 2018, 122, 8286–8300. 10.1021/acs.jpca.8b07491. 30252472

[ref27] Mahoney E. J. D.; Mushtaq S.; Ashfold M. N. R.; Mankelevich Yu.A. Combined Spatially Resolved Optical Emission and Modeling Studies of Microwave-Activated H_2_/Ar and H_2_/Kr Plasmas Operating at Powers and Pressures Relevant for Diamond Chemical Vapour Deposition. J. Phys. Chem. A 2019, 123, 2544–2558. 10.1021/acs.jpca.8b12294. 30852899

[ref28] Mahoney E. J. D.; Rodriguez B. J.; Mushtaq S.; Truscott B. S.; Ashfold M. N. R.; Mankelevich Yu.A. Imaging and Modeling the Optical Emission from CH Radicals in Microwave Activated C/H Plasmas. J. Phys. Chem. A 2019, 123, 9966–9977. 10.1021/acs.jpca.9b08345. 31647649

[ref29] Gore J. P. P.; Mahoney E. J. D.; Smith J. A.; Ashfold M. N. R.; Mankelevich Yu.A. Imaging and Modeling C_2_ Radical Emissions from Microwave Plasma-Activated Methane/Hydrogen Gas Mixtures: Contributions from Chemiluminescent Reactions and Investigations of Higher-Pressure Effects and Plasma Constriction. J. Phys. Chem. A 2021, 125, 4184–4199. 10.1021/acs.jpca.1c01924. 33966382

[ref30] Truscott B. S.; Kelly M. W.; Potter K. J.; Johnson M.; Ashfold M. N. R.; Mankelevich Yu.A. Microwave Plasma Enhanced Chemical Vapour Deposition of Nitrogen Doped Diamond, I: N_2_/H_2_ and NH_3_/H_2_ Plasmas. J. Phys. Chem. A 2015, 119, 12962–12976. 10.1021/acs.jpca.5b09077. 26593853

[ref31] Truscott B. S.; Kelly M. W.; Potter K. J.; Ashfold M. N. R.; Mankelevich Yu.A. Microwave Plasma Enhanced Chemical Vapour Deposition of Nitrogen Doped Diamond, II: CH_4_/N_2_/H_2_ Plasmas. J. Phys. Chem. A 2016, 120, 8537–8549. 10.1021/acs.jpca.6b09009. 27718565 PMC5293323

[ref32] Mahoney E. J. D.; Lalji A. K. S. K.; Allden J. W. R.; Truscott B. S.; Ashfold M. N. R.; Mankelevich Yu.A. Optical Emission Imaging and Modeling Investigations of Microwave Activated SiH_4_/H_2_ and SiH_4_/CH_4_/H_2_ Plasmas. J. Phys. Chem. A 2020, 124, 5109–5128. 10.1021/acs.jpca.0c03396. 32475115

[ref33] Richley J. C.; Fox O. J. L.; Ashfold M. N. R.; Mankelevich Y. A. Combined Experimental and Modeling Studies of Microwave Activated CH_4_/H_2_/Ar Plasmas for Microcrystalline, Nanocrystalline, and Ultrananocrystalline Diamond Deposition. J. Appl. Phys. 2011, 109, 063307 10.1063/1.3562185.

[ref34] Ashfold M. N. R.; Mankelevich Yu.A. Self-Consistent Modeling of Microwave Activated N_2_/CH_4_/H_2_ (and N_2_/H_2_) Plasmas Relevant to Diamond Chemical Vapour Deposition. Plasma Sources Sci. Technol. 2022, 31, 035005 10.1088/1361-6595/ac409e.

[ref35] Krasnoperov L. N.; Kalinovski I. J.; Chu H.-N.; Gutman D. Heterogeneous Reactions of H Atoms and CH_3_ Radicals with a Diamond Surface in the 300–1133 K Temperature Range. J. Phys. Chem. 1993, 97, 11787–11796. 10.1021/j100147a036.

[ref36] Yang X.; Kogut D.; Couëdel L.; Angot T.; Roubin P.; Faure J.-B.; Cartry G. TALIF Measurements of Hydrogen and Deuterium Surface Loss Probabilities on Quartz in Low Pressure High Density Plasmas. Plasma Sources Sci. Technol. 2021, 30, 015013 10.1088/1361-6595/abd454.

[ref37] Gubarev V.; Lopaev D.; Zotovich A.; Medvedev V.; Krainov P.; Astakhov D.; Zyryanov S. Dynamics of H Atoms Surface Recombination in Low-Temperature Plasma. J. Appl. Phys. 2022, 132, 193301 10.1063/5.0119577.

[ref38] Ghahfarokhi P. S.; Kallaste A.; Belahcen A.; Vaimann T. Determination of Heat Transfer Coefficient for the Air Forced Cooling Over a Flat Side of Coil. Electr. Control and Commun. Eng. 2019, 15, 15–20. 10.2478/ecce-2019-0003.

[ref39] Bityukov V. K.; Petrov V. A. Optical Quartz Glass as a Reference Substance for the Thermal Conductivity Coefficient of Partially Transparent Materials. High Temp. 2000, 38, 293–299. 10.1007/BF02755959.

[ref40] Prasanna S.; Rond C.; Michau A.; Hassouni K.; Gicquel A. Effect of Buoyancy on Power Deposition in Microwave Cavity Hydrogen Plasma Source. Plasma Sources Sci. Technol. 2016, 25, 045017 10.1088/0963-0252/25/4/045017.

[ref41] Sneh O.; George S. M. Thermal-Stability of Hydroxyl-Groups on a Well-Defined Silica Surface. J. Phys. Chem. 1995, 99, 4639–4647. 10.1021/j100013a039.

[ref42] Arthur N. L.; Potzinger P. The H/(CH_3_)_3_SiH/C_2_H_4_ Reaction System and the Addition of (CH_3_)_3_Si to Ethene: An End-Product Study. Organometallics 2002, 21, 2874–2890. 10.1021/om011067d.

[ref43] Raghunath P.; Lee Y. M.; Wu S. Y.; Wu J. S.; Lin M. C. *Ab Initio* Chemical Kinetics for Reactions of H Atoms with SiH_*x*_ (*x* = 1–3) Radicals and Related Unimolecular Decomposition Processes. Int. J. Quantum Chem. 2013, 113, 1735–1746. 10.1002/qua.24396.

[ref44] Allendorf M. D.; Melius C. F. Theoretical Study of the Thermochemistry of Molecules in the Si-C-H System. J. Phys. Chem. 1992, 96, 428–437. 10.1021/j100180a080.

[ref45] Grill A.; Sternhagen V.; Neumayer D.; Patel V. Hydrogen Plasma Effects on Ultralow-*k* Porous SiCOH Dielectrics. J. Appl. Phys. 2005, 98, 074502 10.1063/1.2060935.

[ref46] West R.; Baney R. H. Hydrogen Bonding Studies 2. The Acidity and Basicity of Silanols Compared to Alcohols. J. Am. Chem. Soc. 1959, 81, 6145–6148. 10.1021/ja01532a011.

[ref47] Harris G. I. A Study of Hydrogen Bonding in Poly(Diorganosiloxane)-αω-Diols. J. Chem. Soc. 1963, 5978–5982. 10.1039/JR9630005978.

[ref48] Harris G. I. Transmission of Electronic Effects on Organosilanols. J. Chem. Soc. B 1971, 2083–2086. 10.1039/j29710002083.

[ref49] Ketvirtis A. E.; Bohme D. K.; Hopkinson A. C. Theoretical Enthalpies of Formation of Compounds SiCH_*n*_ (*n* = 0–4), SiC_2_H_*n*_ (*n* = 0–4, 6), SiCH_*n*_^+^ (*n* = 0–5), and SiC_2_H_*n*_^+^ (*n* = 0–5,7). J. Phys. Chem. 1995, 99, 16121–16127. 10.1021/j100043a063.

[ref50] Walsh R. Bond Dissociation Energies in Organosilicon Compounds. In Silicon Compounds: Silanes and Silicones (2nd ed.), eds. Arkles B.; Larson G.., Gelest Inc.: 2008, 200–207.

[ref51] Sattelmeyer K. W.; Schaefer H. F.; Stanton J. F. The Global Minimum Structure of SiC_3_: The Controversy Continues. J. Chem. Phys. 2002, 116, 9151–9153. 10.1063/1.1480868.

[ref52] Yang T.; Bertels L.; Dangi B. B.; Li X. H.; Head-Gordon M.; Kaiser R. I. Gas Phase Formation of c-SiC_3_ Molecules in the Circumstellar Envelope of Carbon Stars. Proc. Nat. Acad. Sci. 2019, 116, 14471–14478. 10.1073/pnas.1810370116. 31262805 PMC6642343

[ref53] Sehring C. M.; Palmer C. Z.; Westbrook B. R.; Fortenberry R. C. The Spectral Features and Detectability of Small, Cyclic Silicon Carbide Clusters. Front. Astron. Space Sci. 2022, 9, 1074879 10.3389/fspas.2022.1074879.

[ref54] Apponi A. J.; McCarthy M. C.; Gottlieb C. A.; Thaddeus P. Astronomical Detection of Rhomboidal SiC_3_. Astrophys. J. 1999, 516, L103–L106. 10.1086/311998.

[ref55] Ohishi M.; Kaifu N.; Kawaguchi K.; Murakami A.; Saito S.; Yamamoto S.; Ishikawa S.; Fujita Y.; Shiratori Y.; Irvine W. M. Detection of a New Circumstellar Carbon Chain Molecule, C_4_Si. Astrophys. J. 1989, 345, L83–L86. 10.1086/185558.

[ref56] McElroy D.; Walsh C.; Markwick A. J.; Cordiner M. A.; Smith K.; Millar T. J. The UMIST Database for Astrochemistry 2012. Astron. Astrophys. 2013, 550, A36 10.1051/0004-6361/201220465.

[ref57] McCarthy M. C.; Apponi A. J.; Gottlieb C. A.; Thaddeus P. Laboratory Detection of Five New Linear Silicon Carbides. Astrophys. J. 2000, 538, 766–772. 10.1086/309177.

[ref58] Steglich M.; Maier J. P. Electronic Transitions of Jet-Cooled SiC_2_, Si_2_C_*n*_ (*n* = −1 3), Si_3_C_*n*_ (*n* = 1,2), and SiC_6_H_4_ Between 250 and 710 nm. Astrophys. J. 2015, 801, 119 10.1088/0004-637X/801/2/119.

[ref59] Smith T. C.; Li H. Y.; Clouthier D. J.; Kingston C. T.; Merer A. J. The Electronic Spectrum of Silicon Methylidyne (SiCH), a Molecule with a Silicon–Carbon Triple Bond in the Excited State. J. Chem. Phys. 2000, 112, 3662–3670. 10.1063/1.480518.

[ref60] Butler J. E.; Mankelevich Yu.A.; Cheesman A.; Ma J.; Ashfold M. N. R. Understanding the Chemical Vapour Deposition of Diamond: Recent Progress. J. Phys.: Condens. Matter 2009, 21, 364201 10.1088/0953-8984/21/36/364201. 21832307

[ref61] Wrobel A. M.; Walkiewicz-Pietrzykowska A.; Hatanaka Y.; Wickramanayaka S.; Nakanish Y. Oligomerization and Polymerization Steps in Remote Plasma Chemical Vapor Deposition of Silicon-Carbon and Silica Films from Organosilicon Sources. Chem. Mater. 2001, 13, 1884–1895. 10.1021/cm001044+.

[ref62] Wrobel A. M.; Walkiewicz-Pietrzykowska A.; Uznanski P. Remote Hydrogen Microwave Plasma Chemical Vapor Deposition from Methylsilane Precursors. 1. Growth Mechanism and Chemical Structure of Deposited a-SiC:H Films. Thin Solid Films 2014, 564, 222–231. 10.1016/j.tsf.2014.04.087.

[ref63] Conlin R. T.; Kwak Y. W.; Huffaker H. B. Reactions of 1,1-Dimethylsilene with Alkynes. Organometallics 1983, 2, 343–344. 10.1021/om00074a024.

[ref64] Chernyshev E. A.; Komalenkova N. G. The Thermal Gas-Phase Synthesis of Heterocyclic Compounds with One or Several Silicon Atoms in the Ring. Russ. Chem. Rev. 1989, 58, 559–574. 10.1070/RC1989v058n06ABEH003460.

[ref65] Manion J. A.; Huie R. E.; Levin R. D.; Burgess D. R. Jr.; Orkin V. L.; Tsang W.; McGivern W. S.; Hudgens J. W.; Knyazev V. D.; Atkinson D. B.; et al NIST Chemical Kinetics Database, NIST Standard Reference Database 17, Version 7.0 (Web Version), Release 1.6.8, Data version 2015.09; National Institute of Standards and Technology: Gaithersburg, MD, 20899–8320. https://kinetics.nist.gov/ (accessed 2024–07–20).

[ref66] Mankelevich Yu.A.; Olevanov M. A.; Rakhimova T. V. Dust Particle Coagulation Mechanism in Low-Pressure Plasma: Rapid Growth and Saturation Stage Modeling. Plasma Sources Sci. Technol. 2008, 17, 015013 10.1088/0963-0252/17/1/015013.

[ref67] Goree J. Charging of Particles in a Plasma. Plasma Sources Sci. Technol. 1994, 3, 400–406. 10.1088/0963-0252/3/3/025.

[ref68] Goniakowski J.; Noguera C. Insulating Oxide Surfaces and Nanostructures. C. R. Physique 2016, 17, 471–480. 10.1016/j.crhy.2015.12.007.

[ref69] Cherkashinin G.; Ambacher O.; Schiffer T.; Schmidt G. Mobility Edge in Hydrogenated Amorphous Carbon. Appl. Phys. Lett. 2006, 88, 172114 10.1063/1.2200397.

[ref70] Li M. M.; Jiang L. H.; Sun Y. H.; Xiao T.; Xiang P.; Tan X. Y. Silicon Content Influence on Structure and Photoluminescence Properties of Carbon Rich Hydrogenated Amorphous Silicon Carbide Thin Films. J. Alloys Compd. 2018, 753, 320–328. 10.1016/j.jallcom.2018.04.226.

[ref71] Lukianov A. N.; Klyui N.; Sha B.; Lozinskii V. B.; Temchenko V. P.; Avksentyeva L. V.; Staschuk V. S. Effect of Discharge Power and Silicon Content on Optical and Mechanical Properties of Carbon-Rich Amorphous Silicon Carbide Films Obtained by PECVD. J. Alloys Compd. 2019, 801, 285–294. 10.1016/j.jallcom.2019.06.093.

[ref72] Cho S. H.; Choi D. J. The Study of Dielectric Constant Change of a-SiC:H Films Deposited by Remote PECVD with Low Deposition Temperatures. J. Korean Phys. Soc. 2009, 55, 1920–1924. 10.3938/jkps.55.1920.

[ref73] Popovich A.; Martyanov A.; Khomich A.; Fedotov P.; Savin S.; Sedov V.; Ralchecko V. CVD Diamond-SiC Composite Films: Structure and Electrical Properties. Diamond Relat. Mater. 2022, 125, 108975 10.1016/j.diamond.2022.108975.

[ref74] Smith G. P.; Golden D. M.; Frenklach M.; Moriarty N. W.; Eiteneer B.; Goldenburg M.; Bowman C. T.; Hanson R. K.; Song S.; Gardiner W. C. Jr.; et al GRI-Mech 3.0. Available at http://www.me.berkeley.edu/gri_mech/ (accessed 2024–07–20).

[ref75] Hassouni K.; Mohasseb F.; Bénédic F.; Lombardi G.; Gicquel A. Formation of Soot Particles in Ar/H_2_/CH_4_ Microwave Discharges during Nanocrystalline Diamond Deposition: A Modeling Approach. Pure Appl. Chem. 2006, 78, 1127–1145. 10.1351/pac200678061127.

[ref76] Ouaras K.; Lombardi G.; Hassouni K. Nanoparticle Synthesis in Microwave Plasmas: Peculiarities and Comprehensive Insight. Sci. Rep. 2024, 14, 4653 10.1038/s41598-023-49818-3. 38409179 PMC11231176

[ref77] De Bleecker K.; Bogaerts A.; Goedheer W. Aromatic Ring Generation as a Dust Precursor in Acetylene Discharges. Appl. Phys. Lett. 2006, 88, 151501 10.1063/1.2193796.

[ref78] Janalizadeh S.; Foroutan G.; Foroutan V. Nucleation of Carbonaceous Nanoparticles in a Low Pressure Ar/C_2_H_2_ Plasma with C_2_H_4_ Impurity. J. Phys. Chem. A 2023, 127, 6999–7011. 10.1021/acs.jpca.3c02510. 37556428

[ref79] Smith T. C.; Evans C. J.; Clouthier D. J. Discovery of the Optically Forbidden *S*_1_-*S*_0_ Transition of Silylidene (H_2_C = Si). J. Chem. Phys. 2003, 118, 1642–1648. 10.1063/1.1531618.

[ref80] Kramida A.; Ralchenko Yu.; Reader J. and NIST ASD Team NIST Atomic Spectra Database (version 5.11), [Online]. National Institute of Standards and Technology: Gaithersburg, 2023. Available: https://physics.nist.gov/asd [Sun May 26 2024]. MD. DOI: 10.18434/T4W30F.

[ref81] Ram R. S.; Engleman R. Jr.; Bernath P. F. Fourier Transform Emission Spectroscopy of the A^2^Δ-X^2^Π Transition in SiH and SiD. J. Mol. Spectrosc. 1998, 190, 341–352. 10.1006/jmsp.1998.7582. 9668026

[ref82] Harper W. H.; Waddell K. W.; Clouthier D. J. Jet Spectroscopy, Structure, Anomalous Fluorescence, and Molecular Quantum Beats of Silylidene (H_2_C = Si), the Simplest Unsaturated Silylene. J. Chem. Phys. 1997, 107, 8829–8839. 10.1063/1.475175.

[ref83] Brix T.; Paul U.; Potzinger P. P.; Reimann B. Absorption Spectra of Trialkylsilyl Radicals by a Chemical Modulation Experiment. J. Photochem. Photobiol. A: Chemistry. 1990, 54, 19–25. 10.1016/1010-6030(90)87003-T.

[ref84] Maier G.; Mihm G.; Reisenauer H. P. Silaethene. Angew. Chem., Int. Ed. Engl. 1981, 20, 597–598. 10.1002/anie.198105971.

[ref85] Kerst C.; Boukherroub R.; Leigh W. J. Far-UV Laser Flash Photolysis in Solution. A Study of the Chemistry of 1,1-Dimethylsilene in Hydrocarbon Solvents. J. Photochem. Photobiol. A: Chemistry 1997, 110, 243–246. 10.1016/S1010-6030(97)00194-9.

[ref86] Prij A.; Marsili E.; Hutton L.; Hollas D.; Shchepanovska D.; Glowacki D. R.; Slavíček P.; Curchod B. F. E. Calculating Photoabsorption Cross-Sections for Atmospheric Volatile Organic Compounds. ACS Earth Space Chem. 2022, 6, 207–217. 10.1021/acsearthspacechem.1c00355. 35087992 PMC8785186

[ref87] Hollas D.; Curchod B. F. E. AtmoSpec – a Tool to Calculate Photoabsorption Cross-Sections for Atmospheric Volatile Organic Compounds. J. Phys. Chem. A 2024, 128, 8580–8590. 10.1021/acs.jpca.4c05174. 39359141 PMC11457220

[ref88] Neese F. Software Update: The ORCA Program System - Version 5.0. WIREs Comput. Mol. Sci. 2022, 12, e1606 10.1002/wcms.1606.

[ref89] Reilly N. J.; Steglich M.; Kokkin D. L.; Maier J. P.; Stanton J. F.; McCarthy M. C. Optical Spectrum of Si_3_C, and a Re-Analysis of the C^1^B_1_-X^1^A_1_ Transition. J. Mol. Spectrosc. 2015, 310, 135–140. 10.1016/j.jms.2014.12.025.

[ref90] Lan Y.-Z.; Feng Y.-L. Study of Absorption Spectra and (Hyper)polarizabilities of SiC_*n*_ and Si_*n*_C (*n* = 2–6) Clusters using Density Functional Response Approach. J. Chem. Phys. 2009, 131, 054509 10.1063/1.3195062. 19673576

[ref91] Jiang Z. Y.; Xu X. H.; Wu H. S.; Zhang F. Q.; Jin Z. H. Structure and Stability of Si_*n*_C_*m-n*_ Clusters. J. Mol. Struct. (Theochem) 2002, 589–590, 103–109. 10.1016/S0166-1280(02)00251-8.

[ref92] Li Q.-X.; Lu W.-C.; Zang Q.-J.; Zhao L. Z.; Wang C. Z.; Ho K. M. Carbon-Rich C_9_Si_*n*_ (*n* = 1–5) Clusters from Ab Initio Calculations. Comp. and Theor. Chem. 2011, 963, 439–447. 10.1016/j.comptc.2010.11.010.

[ref93] Lan Y.-Z.; Kang H.-L.; Niu T. One- and Two-Photon Absorptions of the C_*n*_ and C_*n*–1_Si Fullerenes in Gas Phase and Solution. Eur. Phys. J. D 2015, 69, 69 10.1140/epjd/e2015-50789-0.

[ref94] Shi S. L.; Xu S. J.; Wang X. J.; Chen G. H. Theoretical Absorption Spectra of Silicon Carbide Nanocrystals. Thin Solid Films 2006, 495, 404–406. 10.1016/j.tsf.2005.08.222.

[ref95] Wheeler M. D.; Newman S. M.; Orr-Ewing A. J.; Ashfold M. N. R. Cavity Ring-Down Spectroscopy. J. Chem. Soc. Faraday Trans. 1998, 94, 337–51. 10.1039/a707686j.

[ref96] Wrobel A. M.; Walkiewicz-Pietrzykowska A.; Uznanski P. Remote Hydrogen Microwave Plasma Chemical Vapor Deposition from Methylsilane Precursors. 2. Surface Morphology and Properties of Deposited a-SiC:H Films. Thin Solid Films 2014, 564, 232–240. 10.1016/j.tsf.2014.04.089.

[ref97] Dřínuk V.; Strašák T.; Novotný F.; Fajgar R.; Bastl Z. RIR MAPLE Procedure for Deposition of Carbon Rich Si/C/H films. Appl. Surf. Sci. 2014, 292, 413–419. 10.1016/j.apsusc.2013.11.153.

[ref98] Niiranen J. T.; Gutman D. Silicon-Carbon Bond Formation Kinetics: Study of the Reactions of CH_3_ with SiH_3_, Si(CH_3_)_3_ and SiCl_3_. J. Phys. Chem. 1993, 97, 9392–9396. 10.1021/j100139a023.

[ref99] Nagai T.; Smets A. H. M.; Kondo M. Formation of SiH_3_ Radicals and Nanoparticles in SiH_4_–H_2_ Plasmas Observed by Time-Resolved Cavity Ringdown Spectroscopy. Jpn. J. Appl. Phys. 2008, 47, 7032–7043. 10.1143/JJAP.47.7032.

[ref100] See, *e.g*., Tsymbal E. Y. Physics 927, Section 13: Optical Properties of Solids; University of Nebraska. Physics 927 | Evgeny Tsymbal | Nebraska (unl.edu) (accessed 2024–06–24).

[ref101] Racine B.; Ferrari A. C.; Morrison N. A.; Hutchings I.; Milne W. I.; Robertson J. Properties of Amorphous Carbon-Silicon Alloys Deposited by a High Plasma Density Source. J. Appl. Phys. 2001, 90, 5002–5012. 10.1063/1.1406966.

[ref102] Berryman J. G. Random Close Packing of Hard-Spheres and Disks. Phys. Rev. A 1983, 27, 1053–1061. 10.1103/PhysRevA.27.1053.

[ref103] Frenklach M.; Kematick R.; Huang D.; Howard W.; Spear K. E.; Phelps A. W.; Koba R. Homogeneous Nucleation of Diamond Powder in the Gas Phase. J. Appl. Phys. 1989, 66, 395–399. 10.1063/1.343890.

[ref104] May P. W.; Harvey J. N.; Allan N. L.; Richley J. C.; Mankelevich Y. A. Simulations of Chemical Vapor Deposition Diamond Film Growth using a Kinetic Monte Carlo Model and Two-Dimensional Models of Microwave Plasma and Hot Filament Chemical Vapor Deposition Reactors. J. Appl. Phys. 2010, 108, 114909 10.1063/1.3516498.

[ref105] Nikhar T.; Baryshev S. V. Evidence of Gas Phase Nucleation of Nanodiamond in Microwave Plasma Assisted Chemical Vapor Deposition. AIP Adv. 2024, 14, 045334 10.1063/5.0192057.

[ref106] Buschmann V.; Klein S.; Fueß H.; Hahn H. HREM Study of 3C-SiC Nanoparticles: Influence of Growth Conditions on Crystalline Quality. J. Cryst. Growth. 1998, 193, 335–341. 10.1016/S0022-0248(98)00537-5.

[ref107] Mankelevich Yu.A.; Voronina E. N.; Rakhimova T. V.; Palov A. P.; Lopaev D. V.; Zyryanov S. M.; Baklanov M. R. Multi-Step Reaction Mechanism for F Atom Interactions with Organosilicate Glass and SiO_*x*_ Films. J. Phys. D: Appl. Phys. 2016, 49, 345203 10.1088/0022-3727/49/34/345203.

[ref108] Zyulkov I.; Voronina E.; Krishtab M.; Voloshin D.; Chan B. T.; Mankelevich Y.; Rakhimova T.; Armini S.; De Gendt S. Area-Selective Ru ALD by Amorphous Carbon Modification using H Plasma: From Atomistic Modeling to Full Wafer Process Integration. Mater. Adv. 2020, 1, 3049–3057. 10.1039/D0MA00462F.

[ref109] Cottom J.; Hückmann L.; Olsson E.; Meyer J. From Jekyll to Hyde and Beyond: Hydrogen’s Multifaceted Role in Passivation, H-Induced Breakdown, and Charging of Amorphous Silicon Nitride. J. Phys. Chem. Lett. 2024, 15, 840–848. 10.1021/acs.jpclett.3c03376. 38235960 PMC10823530

[ref110] Hao Z.; Huang K.; Deng K.; Sun F.; Liu J.; Chen L.; Mandal S.; Williams O. A.; Li C.; Wang X.; Wei J. Synthesis of Nano-Diamond Film on GaN Surface with Low Thermal Boundary Resistance and High Thermal Conductivity. Carbon 2024, 229, 119491 10.1016/j.carbon.2024.119491.

[ref111] Wei Y.; Li L.; Tsong S. T. Etching of Si(111)-(7 × 7) and Si(100)-(2 × 1) Surfaces by Atomic Hydrogen. Appl. Phys. Lett. 1995, 66, 1818–1820. 10.1063/1.113332.

[ref112] Lin W.; Lv X. Y.; Wang W. L.; Li L.; Zou G. T. Effect of Growth Rate on the Incorporation of Silicon Impurity in Single Crystal Diamond. Mater. Sci. in Semiconductor Processing 2024, 180, 108554 10.1016/j.mssp.2024.108554.

