# Supplementary material for: Reactive Radical Etching of Quartz by Microwave Activated CH4/H2 Plasmas Promotes Gas Phase Nanoparticle Formation
Source: J Phys Chem A. 2024 Dec 10;128(50):10884–905. doi: 10.1021/acs.jpca.4c05787 (PMC11664594; doi:10.1021/acs.jpca.4c05787)
Supplement: Supplementary file 1 — jp4c05787_si_001.pdf [file jp4c05787_si_001.pdf]

Supporting Information to accompany

Reactive Radical Etching of Quartz by Microwave Activated CH_4/H_2 Plasmas Promotes Gas Phase Nanoparticle Formation.

Michael N.R. Ashfold,[1] Basile F.E. Curchod,[1] Daniel Hollas,[1] Jie Ma [2] and Yuri A. Mankelevich.[3]

[1] School of Chemistry, University of Bristol, Bristol, U.K., BS8 1TS.

[2] School of Physics, Sun Yat-sen University, Guangzhou 510275, China;
State Key Laboratory of Optoelectronic Materials and Technologies, Sun Yat-sen University, Guangzhou 510006, China.

[3] Skobeltsyn Institute of Nuclear Physics, Moscow State University, Leninskie gory, Moscow, 119991 Russia.

Computational details

All molecular geometries studied in this work were optimized using the MP2/cc-pVTZ level of theory. Normal mode frequencies were calculated for each molecule to confirm the nature of the critical point on the ground-state potential energy surface. These optimized geometries were used to calculate the vertical excitation energies (VEEs) presented in Table A1 using the EOM-CCSD/aug-cc-pVTZ approach.

UV/visible absorption cross-sections were simulated using the nuclear ensemble approach (NEA) as implemented in the AtmoSpec code.[1] Briefly, the NEA captures the effects of molecular vibrations in the ground state upon electronic transition profiles, without resolving vibrations in the excited electronic states.[2] For each molecule considered, the ground state density was modeled using a Wigner transform for a harmonic vibrational wavefunction at 0 K, using the geometry and normal mode frequencies calculated at the MP2/cc-pVTZ level, from which 500 geometries were sampled. For each geometry, the 10 lowest singlet excited states and corresponding oscillator strengths were determined using EOM-CCSD with a smaller cc-pVDZ basis set for computational expediency. The results obtained with the larger aug-cc-pVTZ basis set (utilized for the values reported in Table A1) are in quantitative agreement with those obtained with the smaller cc-pVDZ basis. Each transition was broadened with a Gaussian function with $\sigma = 0.05$ eV prior to display in Fig. 6. All electronic structure calculations were performed using the ORCA program v5.0.4.[3]

Table SI

Vertical Excitation Energies (VEE, in eV) and Oscillator Strengths (*f*) for Transitions to the First Excited Singlet (S_1) State of *d*-SiC_3, *r*-SiC_3, *l*-SiC_4, *l*-SiC_6 and SiC_3H_2, Computed at the EOM-CCSD/aug-cc-pVTZ Level. The Final Column Reports the Corresponding VEE and *f* Values for the First Higher Excited Singlet Electronic (S_x) State with Appreciable Oscillator Strength. The Last Two Rows Show Data Reported Previously for *l*-SiC_3H and *l*-SiC_4H (from ref. 4).

Species	Ground state	S_1 State symmetry/ VEE / *f*	S_x State symmetry / VEE / *f*
d-SiC_3	$^1A_1$ (C_{2v})	$^1B_1$ 1.90 0.005	$^1B_1$ 3.58 0.025
r-SiC_3	$^1A_1$ (C_{2v})	$^1B_1$ 1.42 0.003	$^1B_1$ 3.93 0.006
l-SiC_4	$^1\Sigma^+$	$^1\Sigma^-$ 2.29 0.000	$^1\Pi$ 3.47 0.007
l-SiC_6	$^1\Sigma^+$	$^1\Sigma^-$ 1.69 0.000	$^1\Pi$ 3.42 0.008
SiC_3H_2	$^1A_1$ (C_{2v})	$^1A_2$ 1.56 0.000	$^1A_1$ 3.69 0.103
l-SiC_3H	$^2\Pi$	$^2\Sigma^+$ 2.19 0.003	$^2\Pi$ 5.36 1.06
l-SiC_4H	$^2\Pi$	$^2\Sigma^-$ 2.66 10^{-5}	$^2\Pi$ 3.18 0.012

[1] Hollas, D.; Curchod, B.F.E. AtmoSpec – a Tool to Calculate Photoabsorption Cross-Sections for Atmospheric Volatile Organic Compounds. *J. Phys. Chem. A* **2024**, *128*, 8580−8590.

[2] Prij, A.; Marsili, E.; Hutton, L.; Hollas, D.; Shchepanovska, D.; Glowacki, D.R.; Slavíček, P.; Curchod, B.F.E. Calculating Photoabsorption Cross-Sections for Atmospheric Volatile Organic Compounds. *ACS Earth Space Chem*. **2022**, *6*, 207-217.

[3] Neese, F. Software Update: The ORCA Program System - Version 5.0. *WIREs Comput. Mol. Sci*. **2022**, *12*, e1606. doi.org/10.1002/wcms.1606.

[4] Kokkin, D.L.; Reilly, N.J.; Fortenberry, R.C.; Crawford, T.D.; McCarthy, M.C. Optical Spectra of the Silicon-Terminated Carbon Chain Radicals SiC_nH (*n* = 3,4,5). *J. Chem. Phys.* **2014**, *141*, 044310.
